# Single-cell RNA-Seq reveals changes in immune landscape in post-traumatic osteoarthritis

**DOI:** 10.3389/fimmu.2022.938075

**Published:** 2022-07-29

**Authors:** Aimy Sebastian, Nicholas R. Hum, Jillian L. McCool, Stephen P. Wilson, Deepa K. Murugesh, Kelly A. Martin, Naiomy Deliz Rios-Arce, Beheshta Amiri, Blaine A. Christiansen, Gabriela G. Loots

**Affiliations:** ^1^ Physical and Life Sciences Directorate, Lawrence Livermore National Laboratory, Livermore, CA, United States; ^2^ School of Natural Sciences, University of California Merced, Merced, CA, United States; ^3^ Department of Orthopaedic Surgery, University of California Davis Health, Sacramento, CA, United States

**Keywords:** osteoarthritis, ptoa, scRNA-seq, immune cells, macrophages, inflammation, knee injury, Trem2

## Abstract

Osteoarthritis (OA) is the most common joint disease, affecting over 300 million people world-wide. Accumulating evidence attests to the important roles of the immune system in OA pathogenesis. Understanding the role of various immune cells in joint degeneration or joint repair after injury is vital for improving therapeutic strategies for treating OA. Post-traumatic osteoarthritis (PTOA) develops in ~50% of individuals who have experienced an articular trauma like an anterior cruciate ligament (ACL) rupture. Here, using the high resolution of single-cell RNA sequencing, we delineated the temporal dynamics of immune cell accumulation in the mouse knee joint after ACL rupture. Our study identified multiple immune cell types in the joint including neutrophils, monocytes, macrophages, B cells, T cells, NK cells and dendritic cells. Monocytes and macrophage populations showed the most dramatic changes after injury. Further characterization of monocytes and macrophages reveled 9 major subtypes with unique transcriptomics signatures, including a tissue resident Lyve1^hi^Folr2^hi^ macrophage population and Trem2^hi^Fcrls^+^ recruited macrophages, both showing enrichment for phagocytic genes and growth factors such as *Igf1*, *Pdgfa* and *Pdgfc.* We also identified several genes induced or repressed after ACL injury in a cell type-specific manner. This study provides new insight into PTOA-associated changes in the immune microenvironment and highlights macrophage subtypes that may play a role in joint repair after injury.

## Introduction

Osteoarthritis (OA) is a severely debilitating joint disease characterized by progressive cartilage loss, bone remodeling, synovial inflammation, and significant joint pain. Historically, OA was considered a purely mechanical disease caused by cartilage wear and tear or injury (1), while rheumatoid arthritis (RA) was deemed a disease caused by excessive joint inflammation (2). However, recent studies have highlighted that inflammation and immune system changes play a role in OA pathogenesis (3–5). Several innate and adaptive immune cell types have been proposed to contribute to OA pathogenesis including macrophages, neutrophils, B cells, T cells and dendritic cells (DCs) (6–9). However, their precise role in OA initiation or progression is still poorly understood. Identifying and characterizing the immune cell subtypes found in the synovial joint during steady state, after injury and during progression to OA will provide insights into disease etiology and treatment options.

Our immune system is a highly complex defense system, activated in response to infection, illness, and injury. Recent studies employing single-cell RNA-sequence (scRNA-seq) analyses of the immune cell infiltrate in various pathological conditions including RA have shed new light on immune cell heterogeneity and their complex functions (10–12). However, only a limited number of studies have attempted to characterize OA-associated immune changes at single-cell level, longitudinally. Recently, using scRNA-seq Chou et al. identified heterogeneous immune populations in human OA synovium including immune regulatory (IR-MΦ) macrophages, inflammatory macrophages (I-MΦ) expressing high levels of class II MHC genes, DCs, B cells, T cells and mast cells (13). This study was performed on patients with advanced OA, all undergoing knee arthroplasty, which prevented the identification of immune changes associated with OA initiation or early stages of joint degeneration. Determining the functional heterogeneity of various immune cell types and elucidating molecular interactions between the immune system and various other cell types in the joint microenvironment that may drive the progressive intra-articular degradation is crucial to understanding OA pathogenesis.

Joint injury is a major risk factor for the development of OA and post-traumatic osteoarthritis and (PTOA) accounts for about 12% of all OA cases (14). Joint injuries including intra-articular fractures and meniscal, ligamentous and chondral injuries have been identified as initiating factors for PTOA (14). Clinical records indicate that more than 50% of individuals with an anterior cruciate ligament (ACL) injury go on to develop PTOA (15). After trauma, an inflammatory response occurs at the site of injury resulting in cellular and molecular changes including synovial cellular infiltration and inflammatory mediator production (16). Transcriptomic analyses in rodent OA models from our group as well as others have revealed the upregulation of many genes encoding for inflammatory cytokines after ACL rupture or surgical destabilization of the medial meniscus (DMM) including *Ccl2*, *Ccl7*, *Il6* and *Il33* (17–19). Inflammatory cytokines have been shown to promote the expression of enzymatic mediators of cartilage degeneration including MMPs and ADAMs, which can lead to adverse tissue remodeling and joint dysfunction (20). It is likely that PTOA is driven by a dysregulated repair and remodeling process. Therefore, delineating injury-induced immune changes in the joint that lead to the production of pro- and anti-degenerative factors is crucial to understand the molecular mechanisms driving progressive joint degeneration after injury and will help us identify therapeutic targets for blunting these unwarranted effects.

In this study, we investigated the immune cell landscape of the knee in a murine injury model at a single-cell resolution and determined injury-induced changes. Knee joint injury was induced in C57BL/6 (BL6) mice using a non-invasive tibial compression (TC) injury model that closely mimics ACL rupture in humans (21). In this model, BL6 consistently show significant cartilage degeneration by 6 weeks post-injury (22–26). Subsequently, we isolated immune cells from knee joints at day 0 [D0: uninjured], D1, D3, D7, D15 and D30 post-injury and profiled using scRNA-seq to characterize the immune compartment in the joint and determine the ACL-injury induced changes. Special emphasis was given to analyzing monocyte and macrophage populations, which significantly expanded after injury. We identified nine monocyte/macrophage subtypes with unique transcriptomic signatures, including a tissue-resident Lyve1^hi^Folr2^hi^ macrophage and a tissue-recruited Trem2^hi^Fcrls^+^ macrophage subpopulation with potential chondro-protective functions, and characterized their gene expression profiles as a function of disease progression. We also examined *Trem2^-/-^* mice using bulk RNA-seq to determine the potential role of Trem2 receptor in macrophage differentiation or function after injury. Additionally, we characterized injury-induced changes in neutrophil and lymphocyte populations. The detailed cellular and molecular level characterization provided in our study will improve the understanding of the functions of distinct immune subtypes and will facilitate identification of new prognostic and therapeutic targets for PTOA.

## Methods

### ACL injury model

10-week-old male C57Bl/6J (BL6) (Jackson Laboratory Bar Harbor, ME, USA; Stock No: 000664) or *Trem2^-/-^* mice (C57BL/6J-*Trem2^em2Adiuj^/*J; Jackson Laboratory Bar Harbor, ME, USA; Stock No: 027197) were subjected to an ACL injury using a non-invasive single dynamic tibial compressive overload model previously described by Christiansen et al. (21). Briefly, the mouse knee was placed between two vertically aligned plates positioned using an electromagnetic material testing system (ElectroForce 3200, TA Instruments, New Castle, DE, USA) and a compressive force was applied (21). ACL rupture occurred after a total compressive force, between 12N-18N, was administered. For pain relief, buprenorphine (0.01 mg/kg) and sterile saline were administered intraperitoneally immediately post-injury. All animal experiments were approved by the Lawrence Livermore National Laboratory and University of California, Davis Institutional Animal Care and Use Committee and conformed to the Guide for the care and use of laboratory animals.

### Histological assessment of the articular joint

Knee joints were collected from uninjured and injured mice at 4-weeks post-injury and processed for histological evaluation as previously described (22). Briefly, whole joints were fixed in 10% Neutral Buffered Formalin (NBF), decalcified using 0.5 M EDTA, and processed for paraffin embedding. Joints were sectioned in the sagittal plane at 6µm and serial medial sections that included the femoral condyles, menisci, and tibial plateaus were prepared for histological assessment of joint tissue integrity. Sections were stained on glass slides using 0.1% Safranin-O (0.1%, Sigma, St. Louis, MO, USA; S8884) and 0.05% Fast Green (0.05%, Sigma, St. Louis, MO, USA; F7252) using standard procedures (IHC World, Woodstock, MD, USA), then imaged using a Leica DM5000 microscope.

### Immunohistochemistry

Sagittal sections from D0, D7, D30 knee joints of BL6 mice were used for IHC. Primary antibodies were incubated overnight at 4°C in a dark, humid chamber following antigen retrieval with Unitrieve (NB325 Innovex Biosciences, Richmond, CA. USA). Secondary antibodies were incubated for 2 hours at room temperature in a dark, humid chamber at 1:500. Negative control slides were incubated with secondary antibody-only. Stained slides were mounted with Prolong Gold with DAPI for nuclei staining (Molecular Probes, Eugene, OR. USA). Slides were imaged using a Leica DM5000 microscope. ImagePro Plus V7.0 Software, QIClick CCD camera (QImaging, Surrey, BC, Canada) and ImageJ V1.53 Software were used for imaging and photo editing. Primary antibodies include: Lyve1 [1:100 (5.5µg/mL); ab218535 Abcam, Cambridge, UK], Trem2 [1:100 (5.0µg/mL); ab95470 Abcam, Cambridge, UK], CD206 [1:100 (10µg/mL); 601431IG, ThermoFisher, Waltham, MA. USA]. Secondary Antibodies include Chicken anti-rabbit 594 (1:500; A21442, Thermofisher, Waltham, MA. USA), Chicken anti-Rabbit 488 (1:500; A21441, ThermoFisher, Waltham, MA. USA), Donkey anti-goat 488 (1:500; A11055, ThermoFisher, Waltham, MA. USA) and Goat anti-Mouse IgG2a 594 (1:500; A21135, ThermoFisher, Waltham, MA. USA).

### Single cell RNA sequencing

Uninjured joints (day 0) and injured joints from 1-, 3-, 7-, 15- and 30 days post-injury (n=5/group) were used for scRNA-seq analysis. Single-cell isolation protocol was optimized to obtain a maximum number of viable cells from the joint without any immune cell contamination from the bone marrow or superficial tissues. Briefly, hindlimbs were dissected free of superficial tissues such as skin and muscle, taking care to maintain the integrity of the knee joint and retaining synovial fluid between tibia and femur. Joint residing cells (cells from cartilage, synovial fluid, synovium, infrapatellar fat pad, fibrous tissue etc.) were liberated by first dissociating the two bones into 7.5 mL of 3% Collagenase 1 solution (Worthington Biochemical, Lakewood, NJ; CLS-1) and 100 µg/mL DNase I (Roche, Basel, Switzerland; 11284932001) in DMEM/F12 shaking at 37°C for two 1-hour digests followed by PBS wash steps between digests. Cells released from the joint were filtered through a 70 μm nylon cell strainer prior to red blood cell (RBC) lysis using ammonium-chloride-potassium (ACK) lysis buffer (ThermoFisher Scientific, Waltham, MA, USA; A1049201). Separation of immune cells was performed using CD45 conjugated magnetic microbeads followed by Miltenyi Biotech MACS separation with LC columns for CD45^+^ cell enrichment. Both immune (CD45^+^) and non-immune (CD45^-^) cell populations were independently sequenced using a Chromium Single Cell 3’ Reagent Kit and Chromium instrument (10X Genomics, Pleasanton, CA). Library preparation was performed according to manufacturer’s protocol and sequenced on an Illumina NextSeq 500 (Illumina, San Diego, CA, USA).

### scRNA-seq data analysis

Raw scRNA-seq data was processed using the 10x Genomics Cell Ranger software (version 6.0.0.) according to manufacturer’s recommended protocols (10X Genomics, Pleasanton, CA, USA). Briefly, raw base call (BCL) files generated by Illumina NextSeq 500 sequencer were demultiplexed into FASTQ files using Cell Ranger ‘mkfastq’. Aligning sequencing data to the mouse reference genome (mm10), barcode counting, and unique molecular identifier (UMI) counting were performed using Cell Ranger ‘count’. Remaining analysis was performed using Seurat R package, which performs quality control and subsequent analyses on the feature-barcode matrices produced by Cell Ranger (27, 28). Output files from Cell Ranger were read into Seurat v3 and cells with fewer than 500 detected genes/cell and genes that were expressed by fewer than 5 cells were filtered out. Dead cells and potential doublets were also excluded from subsequent analysis. Next, the data was normalized by employing a global-scaling normalization method ‘LogNormalize’ and a set of highly variable genes was identified. Then the data from various timepoints were integrated for downstream analysis. After quality control procedures and integration were complete, data was scaled, and the dimensionality of the data was reduced by principal component analysis (PCA). Subsequently, cells were grouped into an optimal number of clusters for *de novo* cell type discovery using Seurat’s ‘FindNeighbors’ and ‘FindClusters’ functions. A non-linear dimensional reduction was then performed *via* uniform manifold approximation and projection (UMAP) and various cell clusters were identified and visualized. Marker genes per cluster were calculated using Seurat’s ‘FindAllMarkers’ function. To characterize various immune cell types in detail, clusters expressing respective cell types-specific markers were extracted and analyzed as described above to identity subtypes and cell type-specific changes. Gene expression plots were created using Seurat’s ‘VlnPlot’, ‘DotPlot’, ‘FeaturePlot’ and ‘RidgePlot’ functions. Genes differentially expressed between two timepoints were identified using ‘FindMarkers’ function. Ontology enrichment analysis was performed using Enrichr (29).

### Pseudo-time trajectory finding

Pseudo-time trajectory of monocytes and macrophages was constructed with Monocle (30). Expression data, phenotype data, and feature data were extracted from the Seurat object and a Monocle ‘CellDataSet’ object was constructed using the ‘newCellDataSet’ function. Highly variable genes from Seurat object were used as ordering genes. Trajectory construction was then performed after dimensionality reduction and cell ordering with default parameters as described before (31).

### Bulk RNA-seq and data analysis

Cells were obtained from D0 and D7 wildtype BL6 and *Trem2^-/-^* mice as described in the scRNA-seq section and were stored at −80°C in RLT lysis buffer supplemented with beta-mercaptoethanol (Qiagen, Hilden. Germany) until processed. Total RNA was isolated using RNeasy^®^ Mini Qiagen kits (Hilden, Germany) and concentrations were determined using a Qubit^®^ RNA HS Assay Kit (ThermoFisher Scientific, Waltham, MA, USA). Quality was assessed before sequencing using an RNA 6000 Nano kit run on an Agilent 2100 Bioanalyzer (Agilent Technologies Inc., Santa Clara, CA, USA). cDNA libraries were prepared from 100 ng of total RNA per manufacturer specification for RNA sequencing using an Illumina mRNA Stranded Library Preparation kit (Illumina Inc., San Diego, CA, USA) and run on an Illumina NextSeq 500 using the High Output 75 cycles kit (Illumina Inc., San Diego, CA, USA).

Raw sequence data generated from Illumina NextSeq500 was de-multiplexed and converted into fastq files using Illumina’s bcl2fastq software. Read quality was assessed using FastQC (32). Reads were then aligned to mouse reference genome mm10 with STAR (33). Subsequently, gene-wise read counts were calculated using ‘featureCounts’ (34). Then data was normalized using TMM normalization method from edgeR (35) and differentially expressed genes were identified using limma and voom (36). Genes with p-value less than 0.05 and fold change greater than 1.25 were considered as significantly differentially expressed genes.

### Flow cytometry analysis

Single cell suspensions were generated as described in the scRNA-seq section then subsequently incubated with the following antibodies at a 1:100 dilution in PBS+1%FBS for macrophage characterization: Biolegend PE/Cyanine7 anti-mouse CD206 (MMR) Antibody (Clone: C068C2), Biolegend PE anti-mouse F4/80 Antibody (Clone: BM8), Biolegend Brilliant Violet 510™ anti-mouse/human CD11b Antibody (Clone: M1/70), Biolegend APC/Cyanine7 anti-mouse CD45 Antibody (Clone: 30-F11), Invitrogen TREM2 Monoclonal Antibody (Clone 78.18), Biolegend Brilliant Violet 510 anti-mouse Ly-6C (Clone: HK1.4), Biolegend APC anti-mouse Ly-6G (Clone: 1A8)and DAPI for viability staining. Flow cytometric analysis was performed on a BD FACSMelody system.

### Statistical analysis

Statistical analyses were performed using R statistical software or GraphPad Prism. One-way ANOVA and post-hoc Tukey’s Test or student’s t-test were used to determine statistically significant differences of mean expression values. Results were considered statistically significant if *p*-value is less than 0.05.

## Results

### Single-cell level characterization of injury-induced immune changes in the mouse knee joint

**Figure 1:**
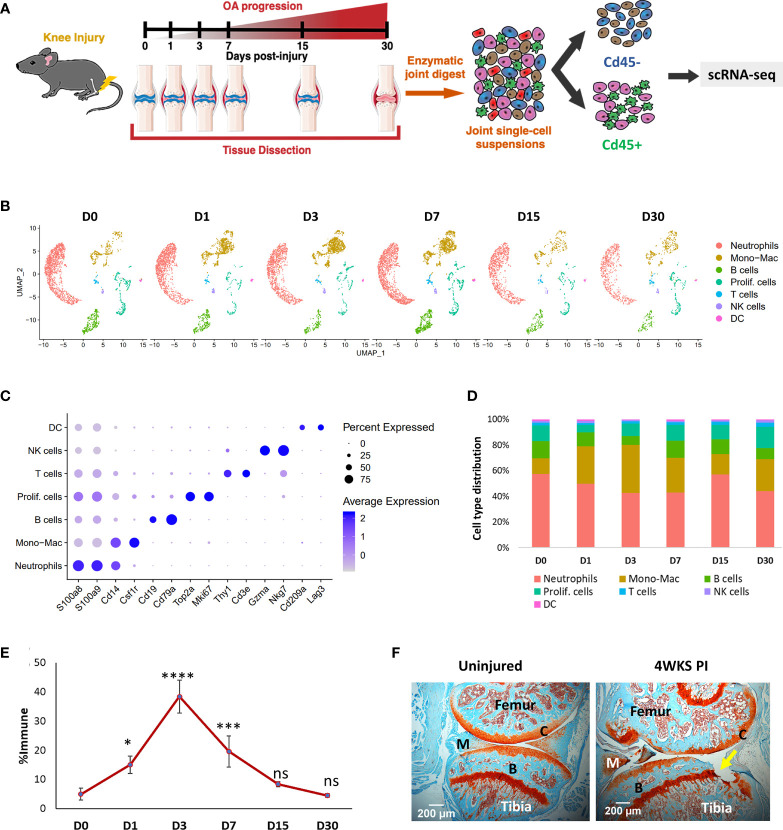
Single-cell analysis of injury-induced immune changes in 10-week-old BL6 mouse knee joints. **(A)** Graphical representation of the experimental workflow. Uninjured (D0) and injured (D1, D3, D7, D15 and D30) knee joints were dissected, dissociated into single cells and immune and non-immune fractions were sequenced separately using scRNA-seq. **(B)** Cell clusters from scRNA-seq analysis visualized by Uniform Manifold Approximation and Projection (UMAP). Colors indicate clusters of various cell types. **(C)** Dot plot showing the expression of selected markers of various cell types. Dot size represents the fraction of cells expressing a specific marker while the intensity of color indicates the average expression level for each gene. **(D)** Changes in the proportion of various immune cell types in the joint over time. scRNA-seq analysis showed a dramatic increase in Mono-Macs after injury, which peaked at D3. **(E)** Flow cytometry analysis of injury-induced changes in monocytes/macrophage population. Compared to D0, there was a significant increase in CD45^+^CD11b^+^Ly6C^+^Ly6G^-^ monocytes/macrophages in the knee joints after injury. Data is represented as mean ± standard deviation (SD). *p<0.01; ***p<0.0001; ****p<0.000001; ns, not significant (one-way ANOVA and Tukey’s post hoc test). **(F)** Safranin-O histological stained sections of uninjured and 4-weeks post-injury joints (4WKS PI). Arrow indicates cartilage damage observed at 4WKS PI. C, cartilage; B, bone; M, meniscus. 5× magnification; scale bars = 200μm.

To identify various synovial joint immune cell types and characterize their dynamic alterations during the pathological progression of PTOA, we profiled the RNA of individual CD45^+^ cells isolated from the knee joints of 10-week-old BL6 mice before ACL injury (D0) and after injury, at various post-injury timepoints (D1, D3, D7, D15 and D30) (**Figure 1A**). Non-immune cells from D0 were also sequenced to investigate potential molecular interactions between immune and connective tissue-forming cells in the joint (**Figure 1A**). Analysis of immune populations identified 7 major cell clusters with distinct gene expression profiles (**Figure 1B**; **S1**). Identity of each cluster was determined based on the expression of previously published cell-type specific markers (**Figure 1B**) (37–40). Cluster 0 was identified as neutrophils based on high expression of *S100a8, S100a9, Mmp9, Retnlg* and *Ly6g* (**Figure 1C**; **S1**). Cluster 1 expressed monocyte/macrophage markers *Cd14*, *Itgam* and *Csf1r* and was labeled ‘Monocyte-Macrophage (Mono-Mac)’. Cells in cluster 2 were identified as B cells based on *Cd19*, *Cd79a* and *Cd79b* expression. Cluster 3 expressed high levels of cell cycle genes *Mki67*, *Cdk1, Stmn1, Top2a* and *Cenpa*; this cluster was annotated as ‘Proliferating cells (Prolif. cells)’ (**Figure 1C**; **S1**). Cluster 4 expressed T cell markers *Cd3e* and *Thy1* while cluster 5 expressed markers for natural killer (NK) cells including *Nkg7* and *Gzma* (**Figure 1C**). Cluster 6 was identified as DCs based on the expression of DC markers *Cd209a* and *Lag3* (**Figure 1C**).

After injury, the most dramatic changes were observed in the Mono-Mac cluster which increased from ~12% of total immune cells sequenced at D0 to ~37.5% at D3 and then started to decrease (**Figure 1D**). Consistent with this, flow cytometry analysis showed an increase in CD45^+^CD11b^+^Ly6C^+^Ly6G^-^ monocyte/macrophages (41) in the joint after injury, which peaked at D3 and then reverted to the D0 levels by D30 (**Figures 1D–E**; **S2**). The proportion of neutrophils and B lymphocytes were elevated by D7, but at D15 the immune composition was very similar to D0 (**Figure 1D**). We observed an increase in the proportion of proliferating immune cells at D30 and by then histologically, the joint started to show visible degeneration (**Figure 1D, F**).

### Monocyte and macrophage compartment changes dramatically after knee injury

**Figure 2:**
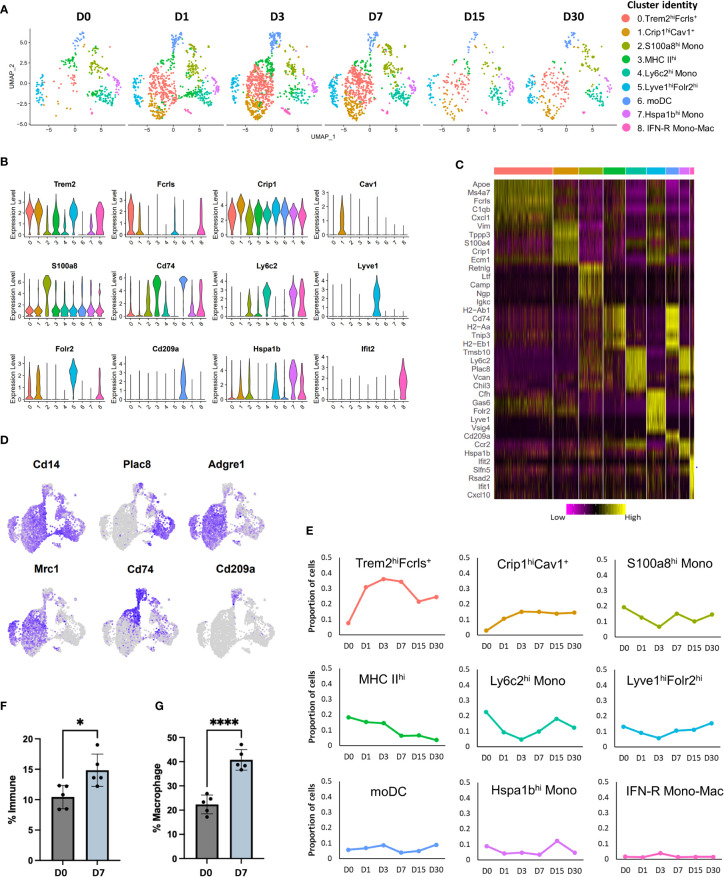
Characterization of injury-induced changes in monocyte/macrophage populations. **(A)** UMAP plots of nine monocyte and macrophage subtypes identified in mouse knee joints at various timepoints. **(B)** Violin plots showing the expression of selected markers of various monocyte and macrophage sub-populations (colored based on cluster identities in panel **(A)**. **(C)** Heatmap showing the scaled expression of top genes enriched in each monocyte/macrophage cluster. **(D)** Feature plots showing the expression of key monocyte and macrophage markers in various clusters. **(E)** Changes in the proportion of various monocyte and macrophage populations after injury, determined using scRNA-seq (colored based on cluster identities in panel **(A)**. **(F)** Flow cytometry analysis of macrophages (CD45^+^CD11b^+^F4/80^+^) in uninjured (D0) and injured (D7) joints. Injured joints at D7 had significantly more macrophages than D0. Data is represented as mean ± SD. *p ≤ 0.05 (two-sided unpaired t-test). **(G)** Flow cytometry data showing the proportion of M2-like (CD45^+^CD11b^+^F4/80^+^ CD206^+^) macrophages at D0 and D7, relative to all macrophages. D7 joints showed a significantly higher proportion of M2-like macrophages compared to D0 joints. Data is represented as mean ± SD. ****p ≤ 0.0001 (two-sided unpaired t-test).

Previous studies have characterized synovial macrophages in steady state and inflammatory arthritis and have shown that they are a highly heterogeneous population with varying functions and origins (11, 42). To determine the cellular diversity within the Mono-Mac cluster (**Figure 1B**) and to identify potential developmental relationships between various macrophages and monocyte subtypes we examined the Mono-Mac cluster in more detail. Sub-clustering Mono-Macs resulted in the identification of 9 clusters with distinct gene expression profiles (**Figures 2A–C**). All clusters expressed *Itgam* (Cd11b) and *Cd14* while *Adgre1* (F4/80) and *Cd68* expression was enriched in clusters 0, 1, 3 and 5 (**Figure 2D**; **S3A, B**), suggesting that these are macrophages. Cluster 0 also showed enrichment for *Trem2*, *Fcrls*, *Ms4a7*, *Apoe, Fabp5, Cd63* and several members of complement pathway (*C1qa*, *C1qb* and *C1qc*) (**Figure 2A-D**; **S3C**); this cluster was annotated as Trem2^hi^Fcrls^+^ macrophages. We also detected robust *Mrc1* (Cd206) expression in this cluster (**Figure 2D**; **S3C**), suggesting that cluster 0 may represent a population of alternatively activated macrophages. Proportion of Trem2^hi^Fcrls^+^ macrophages increased dramatically after injury, peaking at D3 but showed a significant reduction by D15 (**Figure 2E**).

Cluster 1 also shared several genes with Trem2^hi^Fcrls^+^ macrophages including *Trem2*, *Ms4a7* and complement pathway genes *C1qa*, *C1qb* and *C1qc* but had low *Mrc1* expression (**Figures 2A–D**; **S3C**, **Table S1**). In addition, this cluster had significantly higher expression of *Crip1*, *S100a4, Vim, Tagln2* and *Cspg4* than Trem2^hi^ Fcrls^+^ macrophages. Unlike cells in Cluster 0, Cluster 1 macrophages expressed high levels of *Cav1*, a protein known to regulate monocyte differentiation into macrophages (43) and *Aqp1*, a regulator of macrophage migration (44) suggesting that cluster 1 macrophages may represent a pre-activation, early macrophage differentiation stage population (Figure S3D). This cluster was labeled as Crip1^hi^Cav1^+^ macrophages. Similar to Trem2^hi^Fcrls^+^ macrophages, Crip1^hi^Cav1^+^ macrophage population also showed an increase with time (**Figure 2E**).

Cluster 2 showed enrichment for granulocyte-associated transcripts *S100a8*, *Lcn2*, *Retnlg*, *Mmp8* and *Mmp9* along with low to moderate expression of monocyte markers *Ly6c2* and *Plac8* (**Figures 2A-D**; **S3C**, **Table S1**). This cluster also showed enrichment for inflammatory cytokine *Il1b* (**Figure S3E**). Previous studies have described monocytes that express neutrophil markers, suggesting that cluster 2 contains neutrophil-like monocytes (45). This cluster was labelled S100a8^hi^ monocytes. The proportion of S100a8^hi^ monocytes decreased slightly by D3 but reverted back by D7 (**Figure 2E**). Interestingly, a small subset of cells from cluster 2 also expressed B cell markers including *Cd79a* and *Igkc* (**Figure S3F**).

Cluster 3 represented a population of macrophages (*Adgre1* ^+^) showing enrichment for class II MHC genes involved in antigen presentation including *Cd74*, *H2-Aa*, *H2-Ab1* and *H2-Eb1* and this cluster was labeled as MHC II^hi^. (**Figures 2A–D**). Proportion of MHC II^hi^ macrophages gradually decreased after injury (**Figure 2E**). Cluster 6 also showed enrichment for class II MHC genes including *Cd74*, *H2-Aa*, *H2-Ab1* and *H2-Eb1*. This cluster lacked *Adgre1* expression but, expressed *Cd209a*, a marker of monocyte-derived DC (moDC) (**Figure 2D**) (45, 46). Therefore, this cluster was annotated as moDCs. Both these clusters showed significant enrichment for *Il1b* (**Figure S3E**). Cluster 4 showed enrichment for monocyte markers *Ly6c2* and *Plac8* and was identified as Ly6c2^hi^ monocytes (mono) (**Figures 2A–D**). *Il1b* was also enriched in this cluster (**Figure S3E**). Proportion of Ly6c2^hi^ cluster decreased immediately post-injury and then started to increase by D7 (**Figure 2E**). Cluster 7 also expressed monocyte markers *Ly6c2* and *Plac8* but, had significantly higher *Hspa1b*, *Ccr2*, *Tmpo*, and *Rhob* expression and was annotated as Hspa1b^hi^ mono (**Figures 2A–D**; **S3C**).

Cluster 5 contained macrophages (*Adgre1* ^+^) which expressed *Trem2* and several other markers of Trem2^hi^Fcrls^+^ macrophages but also had unique markers including ‘Lymphatic vessel endothelial hyaluronan receptor 1 (*Lyve1*)’, *Folr2*, *Timd4, Sparc* and *Vsig4* (**Table S1**). Robust *Mrc1* (Cd206) expression was also detected in this cluster and this cluster was identified as Lyve1^hi^Folr2^hi^ macrophages (**Figure S3C**). A subset of cells in this cluster expressed *Aqp1*, suggesting the existence of transcriptionally distinct Lyve1^hi^Folr2^hi^ macrophage subtypes (**Figure S3D**). The proportion of Lyve1^hi^Folr2^hi^ macrophages did not change significantly over time (**Figure 2E**). Genes such as *Lyve1*, *Vsig4*, *Timd4* and *Folr2* have previously been identified as markers of a resident macrophage population in multiple tissues (47), suggesting that cluster 5 may represent a synovial tissue resident macrophage population.

Cluster 8 represented a small population of type I interferon responsive (IFN-R) monocytes and macrophages which expressed genes such as *Ifit1*, *Ifit2*, *Ifit3* and *Isg15* (**Figures 2A–C**, **S3C**; **Table S1**). Both monocyte and macrophage marker expression were observed in cluster 8 and this cluster was identified as IFN-R mono-mac. This cluster also expressed high levels of inflammatory cytokines *Cxcl10* (48) and *Ccl12* (49) (**Figure S3E**). Our data showed that there is a dramatic increase in macrophage number after injury which persisted until D7, and a significant proportion of these newly differentiated macrophages acquired an M2-like phenotype (**Figures 2A–E**) consistent with healing. Flow cytometry analysis further confirmed that there are significantly more macrophages (CD45^+^CD11b^+^F4/80^+^) at D7 compared to D0 and that ~40% of macrophages at D7 were M2-like (CD45^+^CD11b^+^F4/80^+^CD206^+^) compared to ~18% M2-like macrophages at D0 (**Figures 2F, G**; **S4**).

**Figure 3:**
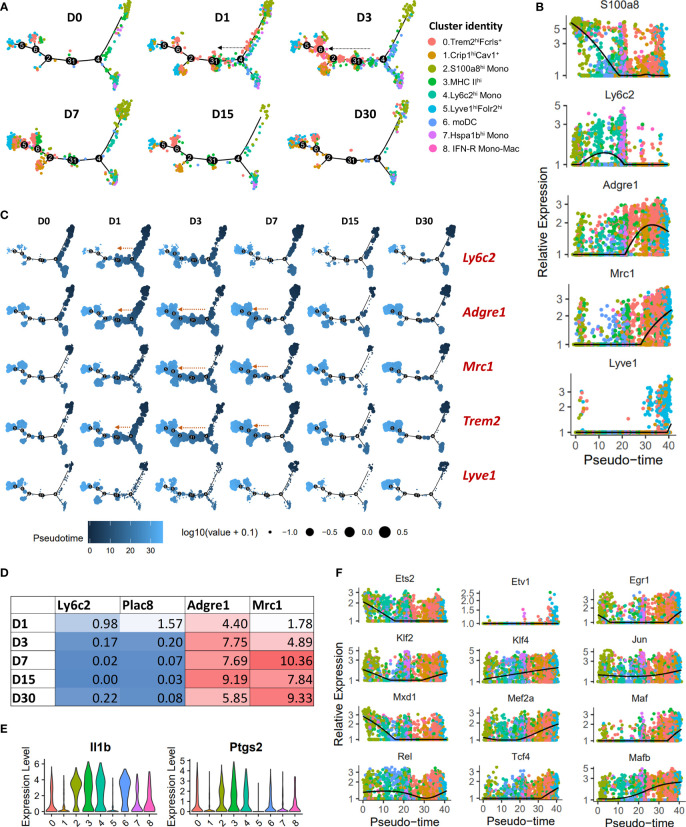
Pseudo-time differentiation trajectory analysis of monocytes and macrophages from D0-D30. **(A)** The relative position of cells across the pseudo-time differentiation trajectory is depicted. Each point is a cell and is colored according to its cluster identity. Cells along the trajectory were divided into six groupings based on experimental timepoints (D0-D30). An expansion of Trem2^hi^Fcrls^+^ population in monocytes → macrophage direction was observed after injury, primarily at D1 and D3 (indicated by arrows). **(B)** Expression of monocyte and macrophage markers on a pseudo-time scale (each point represents a cell and is colored based on cluster identities in panel **(A)**). Monocyte markers were highly expressed at the beginning of the differentiation trajectory while Mrc1 and Lyve1 had the highest expression towards the end. **(C)** Superimposition of the expression of selected genes on the pseudo-time trajectory. Each point is a cell and is colored according to its pseudo-time value. Circle size represents the gene expression level. Expansion of cell populations expressing high levels of Adgre1, Trem2 and Mrc1 in the monocyte → macrophage direction was observed after injury (indicated by arrows). **(D)** Average expression of monocyte and macrophage markers in Trem2^hi^Fcrls^hi^ cluster. **(E)** Violin plots showing elevated Il1b and Ptgs2 expression in MHC II^hi^ macrophages (cluster 3). MHC II^hi^ macrophages and monocytes/moDCs expressed higher levels of Il1b and Ptgs2 compared to other macrophage clusters, including Trem2^hi^Fcrls^+^ and Lyve1^+^ macrophages. **(F)** Plots showing pseudo-time-ordered expression of selected transcription factors (cells are colored based on cluster identities in panel **(A)**).

To determine the potential developmental relationships between various monocyte and macrophage clusters, we performed a pseudo-time differentiation trajectory analysis with Monocle (**Figures 3A, B**; **S5A**). Cells were plotted on the trajectory according to its pseudo-time value which is the inferred distance along a cell’s developmental lineages from the origin of the lineage. Monocytes were found at the start of the pseudo-time trajectory and macrophage populations expressing high levels of *Mrc1* were at the other end (**Figures 3A, B**; **S5A**). S100a8^hi^ monocytes and Hspa1b^hi^ monocytes occupied two separate branches while Ly6c2^hi^ monocytes were distributed on both these branches (**Figure 3A**; **S5A**). We also observed a significant expansion of Trem2^hi^Fcrls^+^ population along the trajectory in a monocyte → macrophage direction at D1-D7 post-injury (**Figure 3A**; **S5A**). This expansion coincided with an increase in *Ly6c2* ^+^ cells primarily at D1, *Adgre1(F4/80)* ^+^ cells at D1-D7, *Mrc1(Cd206)* ^+^ cells primarily at D3-D7 (**Figure 3C**). In addition, we observed an increase in *Adgre1* and *Mrc1* expression and a decrease in the expression of monocyte markers *Ly6c2* and *Plac8* with time in Trem2^hi^Fcrls^+^ cluster (**Figure 3D**). This suggested that Trem2^hi^Fcrls^+^ macrophage population might have originated from Ly6c2+ monocytes and most cells acquire an M2-like phenotype by D3 (**Figures 3A–D**). To further confirm this, we extracted *Ly6c2-*expressing and *Mrc1-*expressing cells from the scRNA-seq data and found that a significant number of cells in Trem2^hi^Fcrls^+^ cluster at D1 and D3 are *Ly6c2* ^+^ cells while a high proportion of cells were *Mrc1* ^+^ at D3-D7 (**Figure S5B**).

MHC II^hi^ macrophages also showed an expansion along the pseudo-time trajectory (**Figure S5A**). This subtype constituted a significant proportion of cells at D0 (**Figure 2E**), suggesting that MHC II^hi^ macrophages could represent the MHCII^+^ tissue resident macrophages described in previous studies (47, 50). They also appeared to be replenished by monocytes after injury; this cluster had Ly6c+ cells at D1 and D3 like Trem2^hi^Fcrls^+^ cluster (**Figure S5B**). We also found elevated levels of *Il1b* and *Ptgs2* expression in cells of MHC II^hi^ cluster, like monocytes (**Figure 3E**). The expression levels were significantly higher than other macrophage clusters, including Trem2^hi^Fcrls^+^, Crip1^hi^Cav1^+^ and Lyve1^+^ macrophages, suggesting that MHC II^hi^ macrophages have an inflammatory phenotype (**Figure 3E**). IFN-R Mono-Mac (*Ifit2*+) showed a slight expansion along the pseudo-time trajectory primarily at D3 indicating increased proliferation/differentiation at this timepoint (**Figures S5A**, **S6A**). We also observed a similar expansion of Crip1^hi^Cav1^+^ macrophages along the trajectory after injury (**Figures S5A**, **S6A**) but, not of Lyve1^+^ macrophages which primarily occupied at the end of the pseudo-time trajectory (**Figure 3C**; **S5A**).

We also identified several transcription factors (TFs) differentially expressed in these monocyte/macrophage subpopulations which may play a role in regulating their unique transcriptomic signatures. By plotting TF expression on a pseudo-time scale, we found that TFs *Mxd1* and *Ets2* had highest expression early in the differentiation trajectory, primarily in monocytes whereas TFs such as *Mafb*, *Mef2a, Mef2c, Klf4* and *Tcf4* had the highest expression towards the end of the pseudo-time trajectory, primarily in *Mrc1*+ macrophage subtypes (**Figure 3F**; **S6B**). *Etv1* and *Maf* were mostly expressed in Lyve1^hi^Folr2^hi^ macrophages whereas *Irf7*, a key regulator of type-I interferon-dependent immune responses (51), was significantly enriched in IFN-R Mono-Mac (**Figure 3F**; **S6B**).

### Lyve1 marks resident macrophages with potential anabolic functions

**Figure 4:**
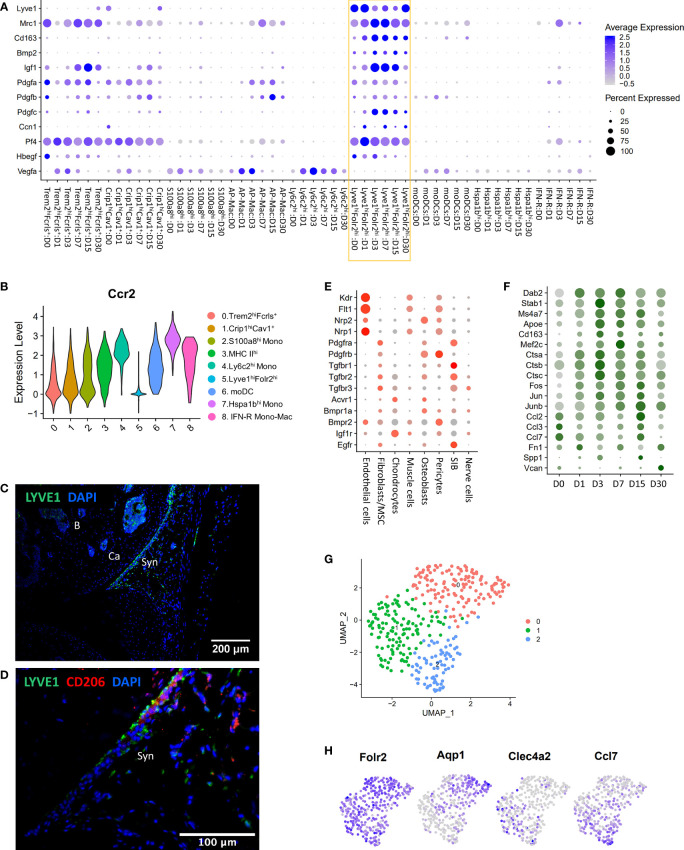
Characterization of Lyve1^hi^Folr2^hi^ macrophages. **(A)** Dot plot showing enrichment of growth factors in Lyve1^hi^Folr2^hi^ macrophages (yellow box). Dot size represents the fraction of cells expressing a specific gene while the intensity of color indicates the average expression level for each gene. **(B)** Violin plot showing the expression of Ccr2 in various monocyte/macrophage subtypes. Lyve1^+^ macrophages showed significantly lower Ccr2 expression. **(C)** IHC analysis of Lyve1+ macrophages at D0 (10× magnification; scale bar = 200μm). Lyve1^+^ macrophages were primarily present at the synovial lining at D0. B: bone; Ca: cartilage; Syn: synovium. **(D)** Co-expression of Lyve1 and CD206 at the synovial lining at D0 (40× magnification; scale bar = 100μm). **(E)** Growth factor receptor expression in connective tissue-forming cells from the joint. Dot size represents the fraction of cells expressing each gene (grey: low expression; red: high expression). **(F)** Genes upregulated in Lyve1^hi^Folr2^hi^ macrophages after injury. Dot size represents the fraction of cells expressing each gene (grey: low expression; green: high expression). **(G)** UMAP plot showing 3 subtypes of Lyve1^hi^Folr2^hi^ macrophages. **(H)** Feature plots showing the expression of Folr2 and markers of various subtypes of Lyve1^hi^Folr2^hi^ macrophages.

**Figure 5:**
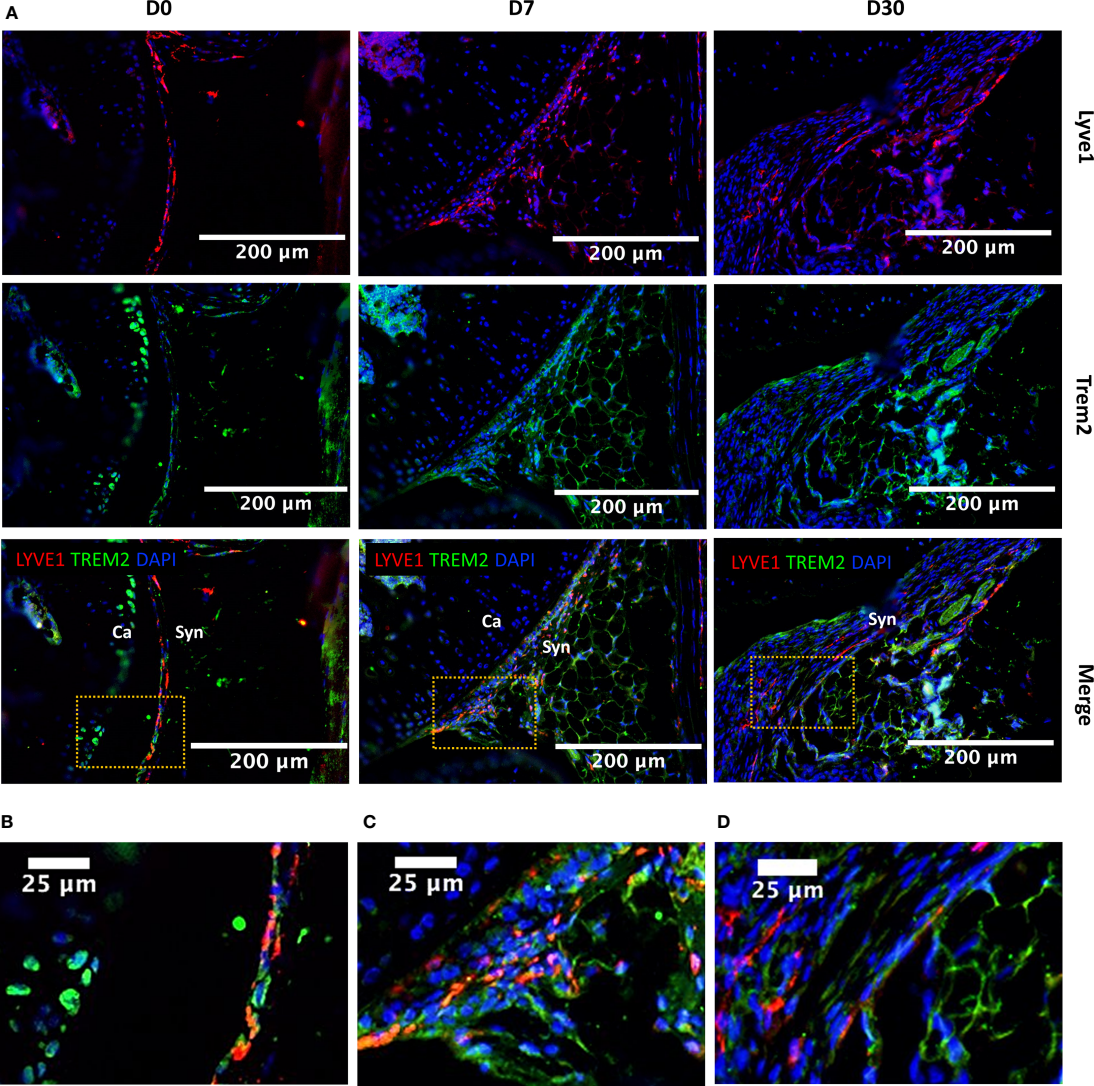
Injury-induced changes in the localization of Lyve1^hi^Folr2^hi^ macrophages based on Lyve1 expression. **(A)** IHC showing Lyve1 and Trem2 expression in macrophages at D0, D7 and D30 (20× magnification; scale bar = 200μm), and a closer view of the images for **(B)** D0, **(C)** D7 and **(D)** D30 matching to the highlighted boxes in A (scale bar = 25μm). The Lyve1^+^ macrophages were primarily observed at the synovial lining at D0. The lining of Lyve1^+^ macrophages was disrupted after injury and these cells started to infiltrate the synovial membrane. Ca, cartilage; Syn, synovium.

Lyve1^hi^Folr2^hi^ macrophages were a major Mono-Mac population at all timepoints examined including D0, and this population robustly expressed *Mrc1* (CD206) and *Cd163*, markers of alternatively activated macrophages (**Figure 4A**). We observed that Lyve1^hi^Folr2^hi^ macrophages did not show an expansion along the differentiation trajectory after injury like other macrophage subtypes, suggesting that they were not actively differentiated from monocytes. Ccr2 expression in macrophages correlates with a monocyte origin and it has been shown that Ccr2^-^ macrophages are not replenished by monocytes (47, 52). We found that Lyve1^hi^Folr2^hi^ macrophages had minimal *Ccr2* expression while all other clusters robustly expressed *Ccr2* (**Figure 4B**). This suggests that Lyve1^hi^Folr2^hi^ cluster may represent self-renewing tissue resident macrophages. IHC analysis showed that Lyve1^high^ macrophages are primarily present at the synovial lining at D0 and express CD206, suggesting that they have an M2-like phenotype (**Figures 4C–D**). The neat lining of Lyve1^+^ macrophages was disrupted, and the spatial orientation of these cells changed after ACL injury in mice (**Figures 5A, B**) and these cells started infiltrating the synovium (**Figures 5A, C, D**). Trem2 receptor expression was also observed in Lyve1+ macrophages (**Figure 5**).

To further understand the potential functions of Lyve1^hi^Folr2^hi^ macrophages in the joint, we examined the gene expression signature of these macrophages and characterized the temporal molecular changes within this population in response to injury. We found that Lyve1^hi^Folr2^hi^ macrophages showed enrichment for several growth factors including *Bmp2*, *Igf1*, *Pdgfa*, *Pdgfb*, *Pdgfc* and *Hbegf* as well as *Ccn1* which also shows growth factor activities (53) (**Figure 4A**). We also observed that this macrophage cluster had extremely low expression for *Vegfa*, an angiogenesis regulating growth factor expressed in several other clusters (**Figure 4A**). Interestingly, Lyve1^hi^Folr2^hi^ macrophages also showed enrichment for *Pf4*, a chemokine commonly considered as megakaryocyte/platelet specific (**Figure 4A**) (54). We observed a significant increase in *Igf1* and *Pdgfc* expression after injury which then started to decrease by D15 (**Figure 4A**). To determine the potential targets of Lyve1^hi^Folr2^hi^ macrophage secreted growth factors, we examined connective tissue forming cells in the joint for growth factor receptor expression and found that chondrocytes had the highest expression of Igf1 receptor *Igf1r* whereas Vegfa receptors *Kdr*, *Flt1*, *Nrp1* and *Nrp2* were primarily expressed in endothelial cells (**Figure 4E**; **S7A**). Bmp2 receptors *Acvr1* and *Bmpr1a* were enriched in osteoblasts, chondrocytes and fibroblasts/MSCs. Hbegf receptor *Egfr* was primarily expressed in fibroblasts/MSCs and synovial intimal fibroblasts (SIB) (**Figure 4E**). Previous studies have identified *Bmp2* and *Igf1* as two cartilage-anabolic cytokines (55–57). *Ccn1* has also been identified as an important regulator of chondrogenesis (53, 58). This suggests that Lyve1^hi^Folr2^hi^ macrophages may have an anabolic function in the joint.

We also identified several genes including *Dab2*, *Cd163*, *Stab1*, *Apoe* and cathepsins *Ctsa*, *Ctsb* and *Ctsc* upregulated in this cluster after injury. The expression of these genes peaked at D3/D7 and was subsequently reduced (**Figure 4F**). *Dab2* is a regulator of inflammatory signaling during phenotypic polarization of macrophages (59). *Stab1* in macrophages has been shown to mediate tissue homeostasis and prevent fibrosis in chronic liver injury (60). *Apoe* deficiency in macrophages results in impaired clearance of apoptotic bodies (61) and cathepsin inhibition led to a polarization shift from M2- to M1 macrophages (62). Several transcription factors including *Fos*, *Jun*, *Junb* and *Mef2c* were also upregulated after injury (**Figure 4F**). We also found that extracellular matrix (ECM) genes including *Fn1*, *Spp1* and *Vcan* were upregulated at D15/D30 (**Figure 4F**). Interestingly, Lyve1^hi^Folr2^hi^ cluster expressed inflammatory cytokines *Ccl2, Ccl3* and *Ccl7* and their expression was reduced after injury and remained low until D7 (**Figure 4F**). Ccl7 is known to be expressed by both M1 and M2 macrophages (63). Park et al. has recently described three vascular macrophage subtypes that shared resident macrophage genes including *Lyve1*, *Mrc1*, *Pf4*, *Cd163*, and *Folr2* and one of which expressed inflammatory and chemokine genes including *Ccl7*, *Ccl2* and *Cxcl1* (64). To further explore the existence of such Lyve1^hi^Folr2^hi^ macrophage subtypes, we extracted all cells from Lyve1^hi^Folr2^hi^ macrophage cluster and reanalyzed using Seurat. Consistent with the findings by Park *et al*, we identified three Lyve1^hi^Folr2^hi^ macrophage subtypes enriched for *Aqp1*, *Clec4a1* and cytokines (*Ccl2*, *Ccl7*), respectively (**Figures 4G, H**; **S7B**) (64). *Clec4a1+ s*ubtype also showed enrichment for transcription actors *Atf3*, *Egr1*, *Jun* and *Fos* (**Figure S7B**). All three clusters expressed *Folr2*, *Lyve1* and *Cd163* (**Figure S7B**) and it is possible that these subclusters represent various differentiation/activation stages of Lyve1^hi^Folr2^hi^ macrophages rather than distinct subtypes.

### Trem2^hi^Fcrls^+^ macrophages assume a molecular profile similar to the resident Lyve1^hi^Folr2^hi^ macrophage population

**Figure 6:**
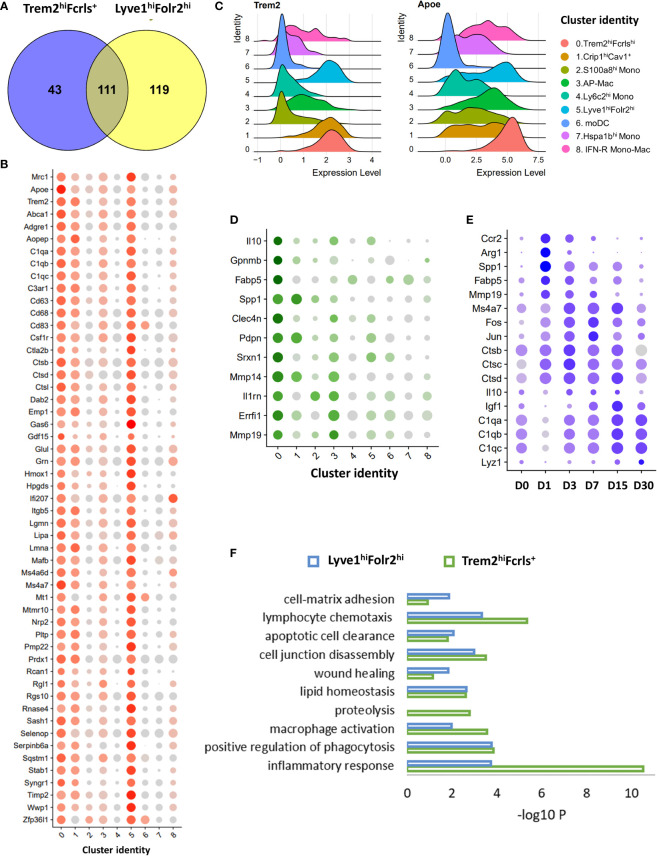
Characterization of Trem2^hi^ M2-like macrophages using scRNA-seq. **(A)** Venn diagrams showing the overlap between genes enriched in both Trem2^hi^Fcrls^+^ and Lyve1^hi^Folr2^hi^macrophages compared to all other clusters. **(B)** Dot plot showing the expression of a subset of genes enriched in both Trem2^hi^Fcrls^+^ and Lyve1^hi^Folr2^hi^macrophages. Dot size represents the fraction of cells expressing each gene (grey: low expression; red: high expression). **(C)** Ridge plot showing the expression of Trem2 and its ligand Apoe in various clusters. Both Trem2 and Apoe were enriched in Trem2^hi^Fcrls^+^ and Lyve1^hi^Folr2^hi^ macrophages. **(D)** Dot plot showing the expression of a subset of genes enriched in Trem2^hi^Fcrls^+^ macrophages compared to Lyve1^hi^Folr2^hi^ macrophages. Dot size represents the fraction of cells expressing each gene (grey: low expression; green: high expression) **(E)** Genes upregulated in Trem2^hi^Fcrls^+^ macrophages after injury. Dot size represents the fraction of cells expressing each gene (grey: low expression; blue: high expression). **(F)** Ontology terms associated with genes enriched in Trem2^hi^Fcrls^+^ and Lyve1^hi^Folr2^hi^macrophages compared to other clusters.

Trem2^hi^Fcrls^+^ macrophages were the major macrophage population in the injured joints until D15 and appeared to be derived from monocyte (**Figures 2A**, **3**). We found that Trem2^hi^Fcrls^+^ macrophages also showed enrichment for *Mrc1* and many of the anabolic growth factors highly expressed in Lyve1^hi^Folr2^hi^macrophages including *Igf1*, *Pdgfa* and *Pdgfb*, suggesting that these two populations might have similar functions (**Figure 4A**; **S7C**). Additionally, we observed Trem2 expression in Lyve1+ synovial macrophages (**Figure 5**). Next, we investigated genes enriched in both these macrophage populations compared to all other monocyte/macrophage clusters. Hundred and fifty-four genes were >1.25 fold enriched (FDR <0.05) in Trem2^hi^Fcrls^+^ cluster compared to all monocytes, MHC II^hi^ and Crip1^hi^Cav^+^ macrophages. Of these, 111 genes were also enriched in Lyve1^hi^Folr2^hi^ cluster (**Figure 6A**). In addition to growth factors, genes common to both Trem2^hi^Fcrls^+^ and Lyve1^hi^Folr2^hi^ macrophages included *Trem2* and its ligand *Apoe* (65), *Dab2*, *Stab1*, *Ms4a7*, cathepsins (*Ctsb*, *Ctsd* and *Ctsl*), complement pathway genes *C1qa*, *C1qb* and *C1qc*, and Mmp inhibitor *Timp2* (**Figures 6B, C**). We also identified many genes enriched in Trem2^hi^Fcrls^+^ macrophages compared to Lyve1^hi^Folr2^hi^ macrophages. These included anti-inflammatory cytokine *Il10, Spp1, Gpnmb, Fabp5, Mmp14 and Mmp19* (**Figure 6D**). Our study also revealed genes activated in Trem2^hi^Fcrls^+^ macrophages at various timepoints post-injury. Genes including *Spp1*, *Ccr2, Fabp5* and *Mmp8* were elevated at D1 while *Igf1* had the highest expression at D7 (**Figure 6E**). *Arg1*, a marker of M2 macrophages was significantly upregulated at D1 and D3 but was dramatically reduced by D7 (**Figures S8A, B**). Similar to Lyve1^hi^Folr2^hi^ macrophages, *Ctsa*, *Ctsb* and *Ctsc* had the peak expression at D3. Complement pathway genes also showed an increase in expression with time (**Figure 6E**). We also observed significant heterogeneity within the Trem2^hi^Fcrls^+^ cluster. A subset of cells showed significant enrichment for transcription factors such as *Jun* and *Fos* while another subset of cells *s*howed enrichment for cytokines such as *Cxcl2* and *Il10* (**Figure S8C**).

Next, we performed an ontology enrichment analysis of genes in Trem2^hi^Fcrls^+^ and Lyve1^hi^Folr2^hi^ macrophages. Both macrophage populations showed enrichment for biological processes such as ‘wound healing’, ‘phagocytosis’, ‘apoptotic cell clearance’ to name a few. (**Figure 6F**). ‘Proteolysis’ was enriched only in the Trem2^hi^Fcrls^+^ cluster, and this cluster also showed higher enrichment for ‘inflammatory response’ (**Figure 6F**). Our data suggests that recruited Trem2^hi^Fcrls^+^ macrophages assume a transcriptional profile similar to Lyve1^hi^Folr2^hi^ macrophages and both these populations may play a role in tissue remodeling *via* phagocytosis and clearing of apoptotic cells after injury. In addition, the growth factors secreted by these macrophage populations might also contribute to tissue repair after injury.

**Figure 7:**
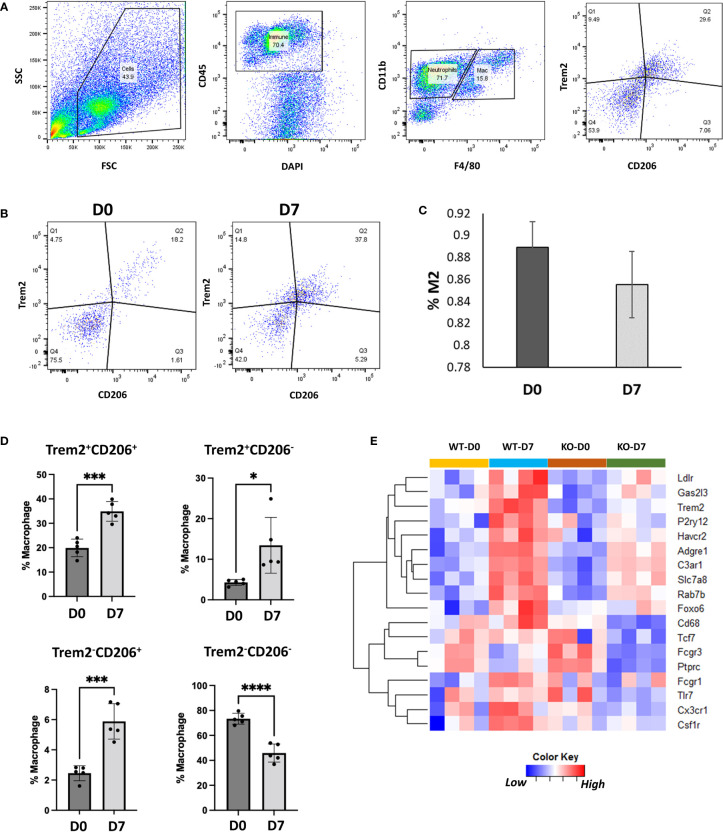
Characterization of Trem2-expressing macrophages using flow cytometry. **(A)** Flow cytometry gating strategy to identify Trem2-expressing M2-like macrophages. **(B)** Flow cytometry plots showing Trem2+ M2-like macrophages at D0 and D7. **(C)** Proportion of Trem2+ macrophages among M2-like macrophages at D0 and D7. Data is represented as mean ± SD. **(D)** Proportion of Trem2 and CD206- expressing macrophages relative to all macrophages. Data is represented as mean ± SD. *p ≤ 0.05; ***p ≤ 0.001; ****p ≤ 0.0001 (two-sided unpaired t-test). **(E)** Genes differentially expressed between injured joints of wildtype (WT) and *Trem2^-/-^* (KO) mice at D7.

TREM2 (triggering receptor expressed on myeloid cells 2) is a cell surface receptor expressed in myeloid lineage cells such as monocytes, macrophages and dendritic cells and microglia in brain (66, 67). We found that *Trem2* and its ligand *Apoe* are robustly expressed in M2-like Trem2^hi^Fcrls^+^ and Lyve1^hi^Folr2^hi^ macrophage populations (**Figure 6B**). Trem2 and Apoe have previously been shown to play a role in phagocytosis (61, 68) and Trem2+ macrophages have been implicated in tissue repair responses in multiple organs (69, 70). To confirm Trem2 expression in M2-like macrophages we performed flow cytometry analysis of synovial macrophages from D0 and D7. Flow cytometry analysis showed that >85% of the M2-like macrophages (CD45^+^CD11b^+^F4/80^+^CD206^+^) in the joint express Trem2 (**Figures 7A–C**). Consistent with scRNA-seq data, injured joints at D7 had significantly more Trem2^+^ M2-like macrophages compared to uninjured D0 joints while macrophages from D0 joints were primarily Trem2^-^CD206^-^(**Figure 7D**). To determine if Trem2 plays a significant role in M2 macrophage differentiation, we analyzed macrophages from uninjured and injured joints of wildtype (WT) and *Trem2* deficient mice (*Trem2^-/-^*) using flow cytometry. The proportion of M2 macrophages was not significantly different between these mice (**Figure S8D**), however bulk RNA-seq analysis of cells from uninjured and injured joints of WT and *Trem2^-/-^* mice determined that *Trem2^-/-^* mice had significantly lower expression of *Cx3cr1*, *Ptprc*, *Adgre1*, *Csf1r*, *Havcr2*, *Ldlr*, *P2y12, Fcgr1* and *Fcgr3* at D7 compared to WT (**Figure 7E**). *Fcgr1*, *Fcgr3* and *P2y12* play roles in phagocytosis (71, 72) while genes such as *Cx3cr1* and *Csf1r* are involved in macrophage differentiation, recruitment or activation (73–75). Downregulation of these genes in *Trem2^-/-^* mice indicates an altered macrophage response after injury and suggests that Trem2 signaling may play a major role in joint repair by modulating macrophage activation and phagocytosis.

### Characterization of neutrophils and lymphocytes

**Figure 8:**
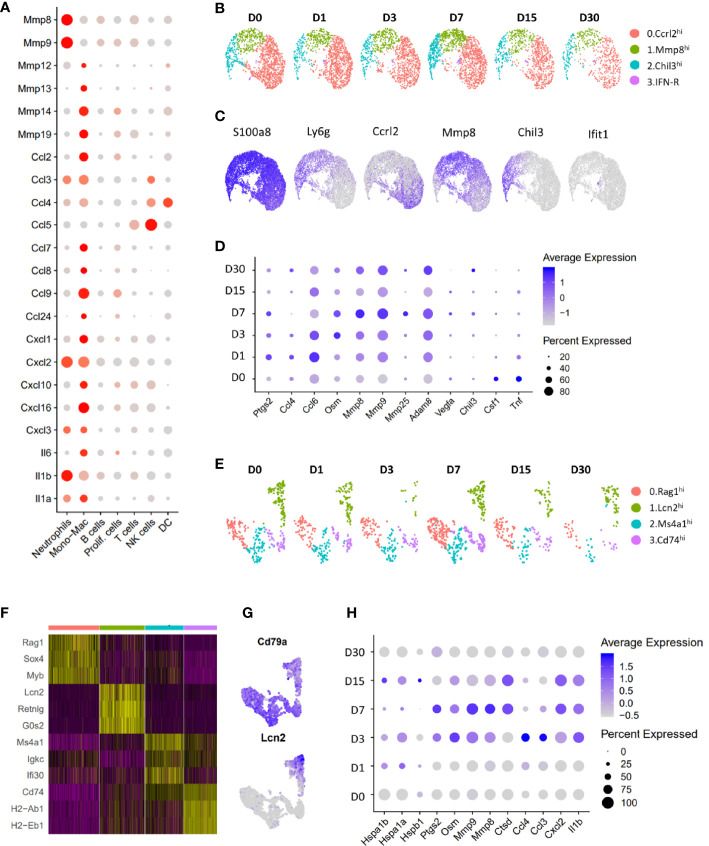
Characterization of neutrophils and B lymphocytes. **(A)** Dot plot showing the expression of cytokines and proteases in various immune cell clusters. Dot size represents the fraction of cells expressing a specific marker while the intensity of color indicates the average expression level for each gene (grey: low expression; red: high expression). **(B)** UMAP plot showing various neutrophil sub-clusters. **(C)** Feature plot showing the expression of various neutrophil sub-cluster markers as well as pan-neutrophil marker S100a8. **(D)** Dot plot showing genes differentially expressed in neutrophils after injury. Dot size represents the fraction of cells expressing a specific marker while the intensity of color indicates the average expression level for each gene. **(E)** UMAP plot showing various B cell sub-clusters. **(F)** Heatmap showing genes enriched in each B cell subtype. **(G)** Feature plot showing the expression of Lcn2 and B cell marker Cd79a in B cell subtypes. **(H)** Genes differentially expressed in Lcn2^hi^ B cell-like cells after injury. Dot size represents the fraction of cells expressing a specific marker while the intensity of color indicates the average expression level for each gene.

Neutrophils were the most abundant immune population identified at all timepoints examined (**Figures 1B–D**) and appeared to be a major source of matrix degrading enzymes such as *Mmp8* and *Mmp9* and several cytokines including *Il1a*, *Il1b*, *Cxcl2*, *Cxcl3* and *Ccl3* in the joints (**Figure 8A**). Further analysis of cell from the neutrophil cluster revealed 4 major subtypes with distinct gene expression profiles: 1) Ccrl2^hi^ neutrophils expressing high levels of *Ccrl2*, *Il1b*, *Ptgs2* and *Cxcl2*; 2) Mmp8^hi^ neutrophils were enriched for *Mmp8*, *Mmp9*, *Retnlg* and *Ly6c2*; 3) Chil3^hi^ neutrophils had high expression of *Chil3*, *Ngp* and *Lcn2* and 4) Interferon (Ifn)-responsive neutrophils expressed high levels of *Ifit1* and *Isg15* (**Figures 8B, C**). We also identified several genes up regulated in the neutrophil cluster after injury including Oncostatin M (*Osm*), *Ptgs2*, *Mmp8*, *Mmp9* and *Adam8* (**Figure 8D**).

T, NK and DC clusters were underrepresented in our data which prevented us from identifying significant injury induced changes in these cell types. Analysis of B cell cluster revealed four subtypes of B cells, all expressing *Cd79a* (**Figures 8E–G**). Interestingly, one of the B cell subtypes expressed granulocyte markers *Lcn2*, *Retnlg* and *Mmp8* and was label as Lcn2^hi^ B cell-like cells (**Figures 8E–G**). We found a significant increase in inflammatory cytokine and matrix-degrading enzyme expression in this cluster after injury (**Figure 8H**), suggesting that Lcn2^hi^ B cell-like cells may also contribute to PTOA pathogenesis.

## Discussion

Using scRNA-seq technology to unbiasedly investigate how injury alters the synovial joint immune compartment, our study revealed a heterogeneity of transcriptional responses within various immune populations including macrophages, neutrophils, and B cells, not previously appreciated by traditional bulk RNA-seq or other gene expression profiling approaches. Neutrophils were identified as a major source of inflammatory cytokines including *Il1a* and *Il1b* and matrix degrading enzymes including *Mmp8*, *Mmp9* and *Adam8* (**Figure 8A**), which indicates that persistence of these cells in the joint, post injury may significantly influence PTOA development. We also observed a significant number of B cells in the joint. Interestingly, we identified a B cell subtype expressing both B cell and granulocyte markers (**Figures 8E–H**). This cluster also showed an increase in cytokine and protease expression after injury and may also contribute to OA pathogenesis. Studies have shown that B cells can differentiate into granulocyte-macrophage progenitor-like cells (76). However, further studies are required to determine the true identity and role in OA pathogenesis of these immune cell subtypes.

Monocyte/macrophage cluster showed the most dramatic changes after injury (**Figures 1D, E**). Monocytes and macrophages play critical roles in tissue repair after injury by regulating inflammation, removing debris and dead cells, and producing cytokines and growth factors that promote healing (77). Our analysis uncovered nine transcriptionally distinct monocytes/macrophages populations and defined injury-induced changes in these populations. One of the major populations we identified is a tissue resident Lyve1^hi^Folr2^hi^ macrophage cluster that robustly expressed genes such as *Lyve1*, *Folr2*, *Timd4, Sparc* and *Vsig4* along with M2 macrophage markers *Mrc1* and *Cd163.* Previous studies identified Lyve1^hi^ tissue-resident macrophages in multiple organs, including heart, lungs, bladder, blood vessels and fat tissues and suggested that these macrophages play a key role in healing and tissue repair (78–81). Consistent with previous studies, Lyve1^hi^Folr2^hi^ macrophages appeared to be self-renewing (47, 50) and not be recruited to the site of injury from other parts of the body. Chakarov *et al.* recently showed that lack of Lyve1^+^ macrophages exacerbated lung fibrosis (80) while Dick *et al.* showed that Lyve1^+^ resident cardiac macrophages have a protective role after myocardial infarction (50). Culemann et al. described a distinct population of synovial tissue-resident macrophages which co-express genes such as *Vsig4, Trem2* and *Sparc* and form an internal immunological barrier at the synovial lining (42). It has also been shown that the Folr2^high^Lyve1^pos^ macrophage population is reduced in patients with treatment-naïve and -resistant RA compared to healthy individuals and it is increased in RA patients under remission (11). Our results are consistent with these previous studies and indicate that Lyve1^hi^Folr2^hi^ macrophages reside primarily at the synovial lining and are involved in tissue repair and remodeling after injury. We found that Lyve1^hi^Folr2^hi^ macrophages express *Bmp2* and *Igf1* and since Igf1 receptor *Igfr* is highly expressed on the surface of chondrocytes while Bmp2 receptor *Acvr1* is enriched in chondrocytes and osteoblasts, we postulate that the secretion of these growth factors by these macrophages promote cartilage growth and repair, consistent with the chondro-protective role of these growth factors in the knee (55–57).

We also identified a subpopulation of macrophages, Trem2^hi^Fcrls^+^, that we hypothesize to be recruited into the joint, post injury, and to proceed to adopt a transcriptional signature similar to the Lyve1^hi^Folr2^hi^ macrophage, further aiding in the healing process. This population was dramatically increased after injury. Our data suggest that these macrophages originate from Ly6c^+^ monocytes. We also identified several transcription factors including *Maf, Mafb*, *Mef2a, Mef2c, Klf4, Etv1* and *Tcf4* that may play a role in the differentiation/function of these macrophages (**Figure 3F**). *Maf*, *Mafb* and *Klf4* have previously been shown to promote M2 polarization (82–84). Etv1 has previously been identified as a transcription factor enriched in Lyve1^hi^ macrophages and has been implicated in macrophage polarization (85, 86).

Both Lyve1^hi^Folr2^hi^ and Trem2^hi^Fcrls^+^ macrophage populations showed enrichment for genes including *Trem2*, its ligand *Apoe*, complement genes (*C1qa*, *C1qb* and *C1qc*) and Cathepsins (*Ctsb*, *Ctsd* and *Ctsl*) and multiple growth factors. However, resident macrophage markers such as *Lyve1*, *Vsig4*, *Timd4* and *Folr2* were not adopted by Trem2^hi^Fcrls^hi^ macrophages. Trem2 is a transmembrane receptor which has been shown to bind to high-density lipoproteins (HDL), low-density lipoproteins (LDL), and several apolipoproteins, including *Apoe* (65). Trem2 enhances the phagocytosis function of microglia and suppress neuroinflammation; in humans, *TREM2* loss-of-function variants are associated with Alzheimer’s disease (65, 87). Several prior studies have implicated Trem2^+^ macrophages in tissue repair (69, 88) and Trem2 has been described as a promotor of macrophage phenotypic switching during tissue repair, tuning down the recruited macrophage inflammatory profile (69). Furthermore, it has been shown that Trem2^hi^ macrophages were reduced in RA synovium compared to healthy synovium, and patients undergoing RA remission showed an increase in Trem2^hi^ macrophages (11). This correlation further stresses the potential protective role of Trem2+ macrophages, in the joint. We also found that >85% of alternatively activated macrophages in the injured knee joints are Trem2+. Injured joints from *Trem2^-/-^* mice had significantly lower levels of genes involved in macrophage differentiation, recruitment, activation or phagocytosis such as *Cx3cr1*, *Csf1r*, *Fcgr1*, *Fcgr3* and *P2y12* play roles in phagocytosis (71–75), suggesting that Trem2 plays a major role in regulating macrophage responses after injury. Future studies will provide more insights into the functions of Trem2+ macrophages in the synovial joint, both in steady state and after injury.

**Figure 9:**
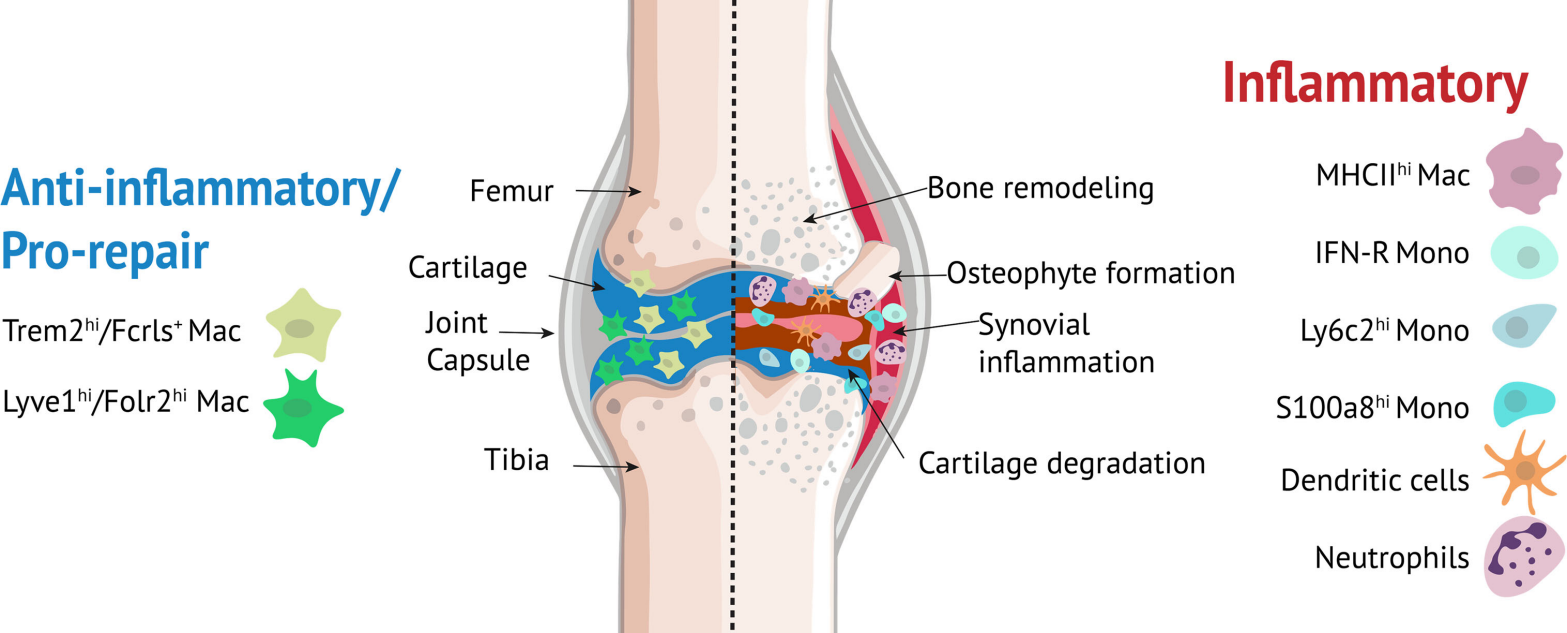
Myeloid cells in the knee joint. Lyve1^hi^Folr2^hi^ and Trem2^hi^Fcrls^+^ macrophages play an anti-inflammatory/pro-repair role while monocytes, moDCs, MHC II^hi^ macrophages and neutrophils may have pro-inflammatory roles.

Our studies also identified additional monocyte/macrophage populations including Ly6c+ monocytes, interferon-responsive cells (IFN-R mono-mac), Crip1^hi^Cav1^+^ macrophages, MHC II^hi^ macrophages, granulocyte-like monocytes (S100a8^hi^) and moDCs. It remains to be determined whether all nine monocyte/macrophage clusters found in knee joints are distinct subpopulations or some merely represent a transcriptional state during differentiation. We observed that a subset of cells from S100a8^hi^ cluster expressed B cell markers. It has previously been shown that pre/pro-B cells can generate macrophage populations suggesting that these Cd79a+ cells may have originated from B cells (89). However, further studies are required to confirm the origin of these cells. Monocyte clusters, moDCs and MHC II^hi^ macrophages showed significant enrichment for *Il1b*, an inflammatory cytokine involved in OA (90) while IFN-R mono-mac expressed high levels of *Cxcl10* and *Ccl12*. Cxcl10 plays a role in regulating neutrophil-NK cell cross-talk determining the severity of experimental OA (91) and macrophage secretion of *Ccl12* has been shown to inhibit fibroblast-mediated cardiac wound healing (49). It is likely that Lyve1^hi^Folr2^hi^ and Trem2^hi^Fcrls^+^ macrophages play a chondro-protective role and promote the healing process (92, 93) after joint injury while monocytes, moDCs, MHC II^hi^ macrophages and neutrophils may have pro-inflammatory functions (**Figure 9**). Additional studies are required to understand the specific functions of various monocyte/macrophage subtypes in the joint and the cues that drive differentiation of monocytes to a specific fate.

Our study highlighted several monocyte/macrophage subpopulations that might play significant roles in orchestrating the response to ACL injury. We hypothesize that these immune subpopulations may also play a role in other types of joint injuries including a tibial plateau fracture or a meniscal tear as changes in these monocyte/macrophage subpopulations have also been identified in other tissues after injury and in inflammatory diseases (11, 12, 94). Dysregulated innate immune response is likely a key contributing factor to pathological changes occurring in the joint after injury including synovial inflammation, cartilage degeneration, abnormal bone remodeling and osteophyte formation (**Figure 9**) and early PTOA progression may be prevented or delayed by interventions with anti-inflammatory/immune modulatory drugs, including the ones currently in use for the treatment of RA (95). Our study identified multiple monocyte/macrophage subtypes that could be potentially targeted to prevent or treat PTOA. However, a thorough understanding of the precise functions of these monocyte/macrophage subpopulations is necessary to effectively target these populations with minimal side effects. Studies focused on detailed molecular and functional characterization of some of these macrophage subtypes in the context of PTOA are currently underway. Findings from these studies could pave the way for the development of novel therapeutic strategies including cell-based therapies for OA such as modulating patient’s own immune cells to prevent or slowdown joint degeneration after injury.

While we were able to identify multiple neutrophil, monocyte/macrophage and B cell subpopulations, we failed to characterize the heterogeneity in T/NK cells and DCs due to underrepresentation of these cell types in our data. There is also a possibility that we missed some neutrophil, monocyte/macrophage and B cell subpopulations present in the joints. As single-cell isolation protocols improve, we will be able to release more viable cells from mineralized tissues such as bone and cartilage and sequence much higher number of cells, which will allow us to characterize rare and novel cell populations. Our study highlights the tremendous complexity and plasticity that underlie the synovial joint immune compartment in steady state, and after ACL injury. Our findings also provide insights into the potential roles of some of these immune subtypes in the joint. A greater understanding of the differential contribution of individual immune subsets to maintaining synovial joint homeostasis and joint tissue repair after injury will open new avenues for developing improved therapeutic strategies for PTOA and other joint diseases.

## Data availability statement

The datasets presented in this study can be found in online repositories. The name of the repository and accession numbers can be found below: NCBI GenBank (https://www.ncbi.nlm.nih.gov/genbank); GSE200842 and GSE200843

## Ethics statement

The animal study was reviewed and approved by Lawrence Livermore National Laboratory IACUC and UC Davis IACUC.

## Author contributions

Conceptualization: AS and GL. Methodology: AS, JM, NH, DM, KM, BC, GL, BA and NR. Formal analysis, AS, NH, SW. Writing- original draft: AS, GL. Writing—review & editing: NH, SW, JM, BC. Project management: AS and GL. All authors contributed to the article and approved the submitted version.

## Funding

This study received funding from DOD PR192271, LDRD 2020 20-LW-002 and DOD PR180268.

## Acknowledgments

This work was performed under the auspices of the U.S. Department of Energy by Lawrence Livermore National Laboratory under Contract DE-AC52-07NA27344.

## Conflict of interest

The authors declare that the research was conducted in the absence of any commercial or financial relationships that could be construed as a potential conflict of interest.

## Publisher’s note

All claims expressed in this article are solely those of the authors and do not necessarily represent those of their affiliated organizations, or those of the publisher, the editors and the reviewers. Any product that may be evaluated in this article, or claim that may be made by its manufacturer, is not guaranteed or endorsed by the publisher.
