# Supplementary material for: Single-cell RNA-Seq reveals changes in immune landscape in post-traumatic osteoarthritis
Source: Front Immunol. 2022 Jul 29;13:938075. doi: 10.3389/fimmu.2022.938075 (PMC9373730; doi:10.3389/fimmu.2022.938075)
Supplement: Supplementary file 1 [file DataSheet_1.docx]

Supplementary Material


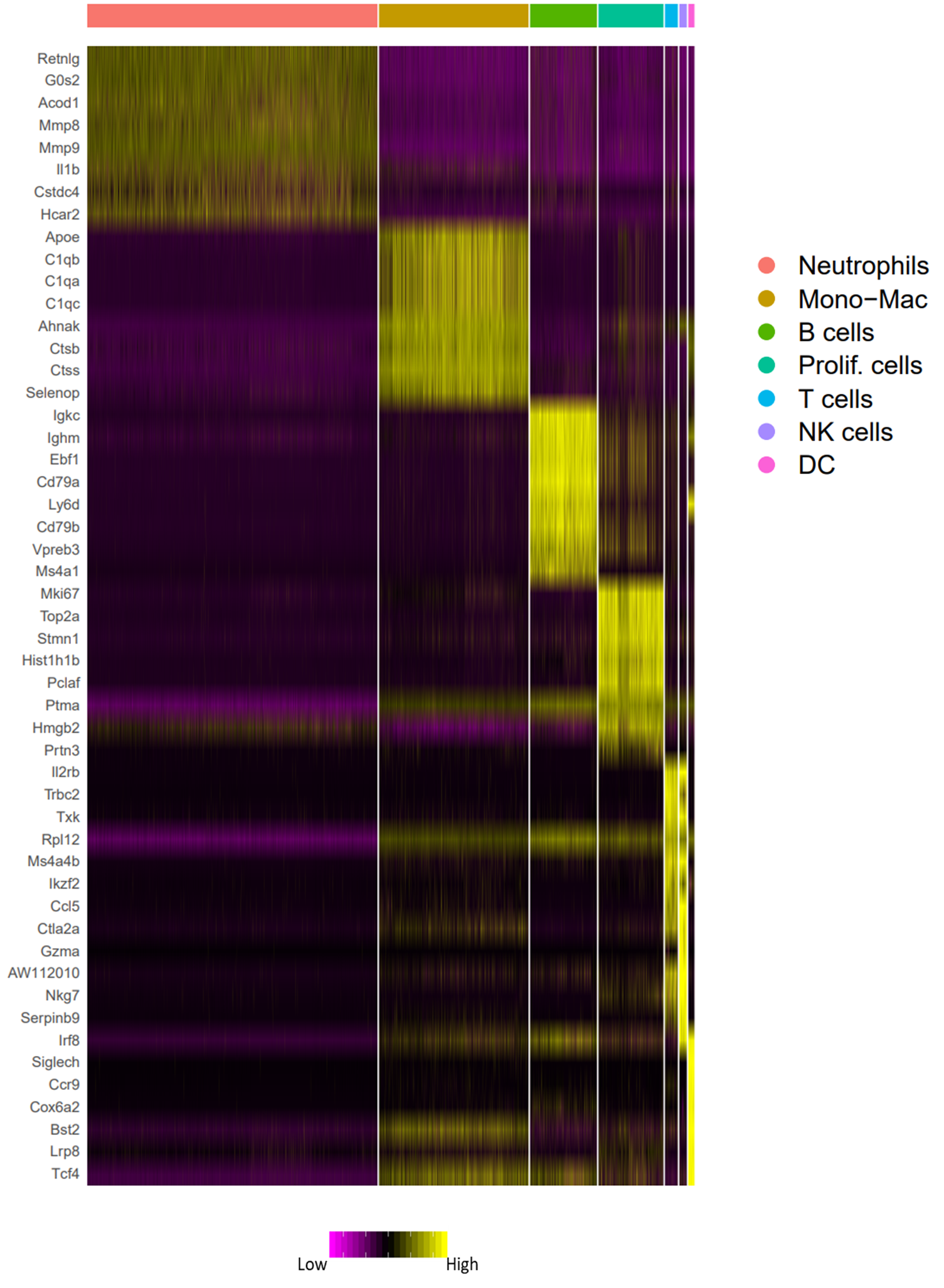


***Figure S1****. Heatmap showing genes enriched in various immune cell types identified in the mouse knee joints.*


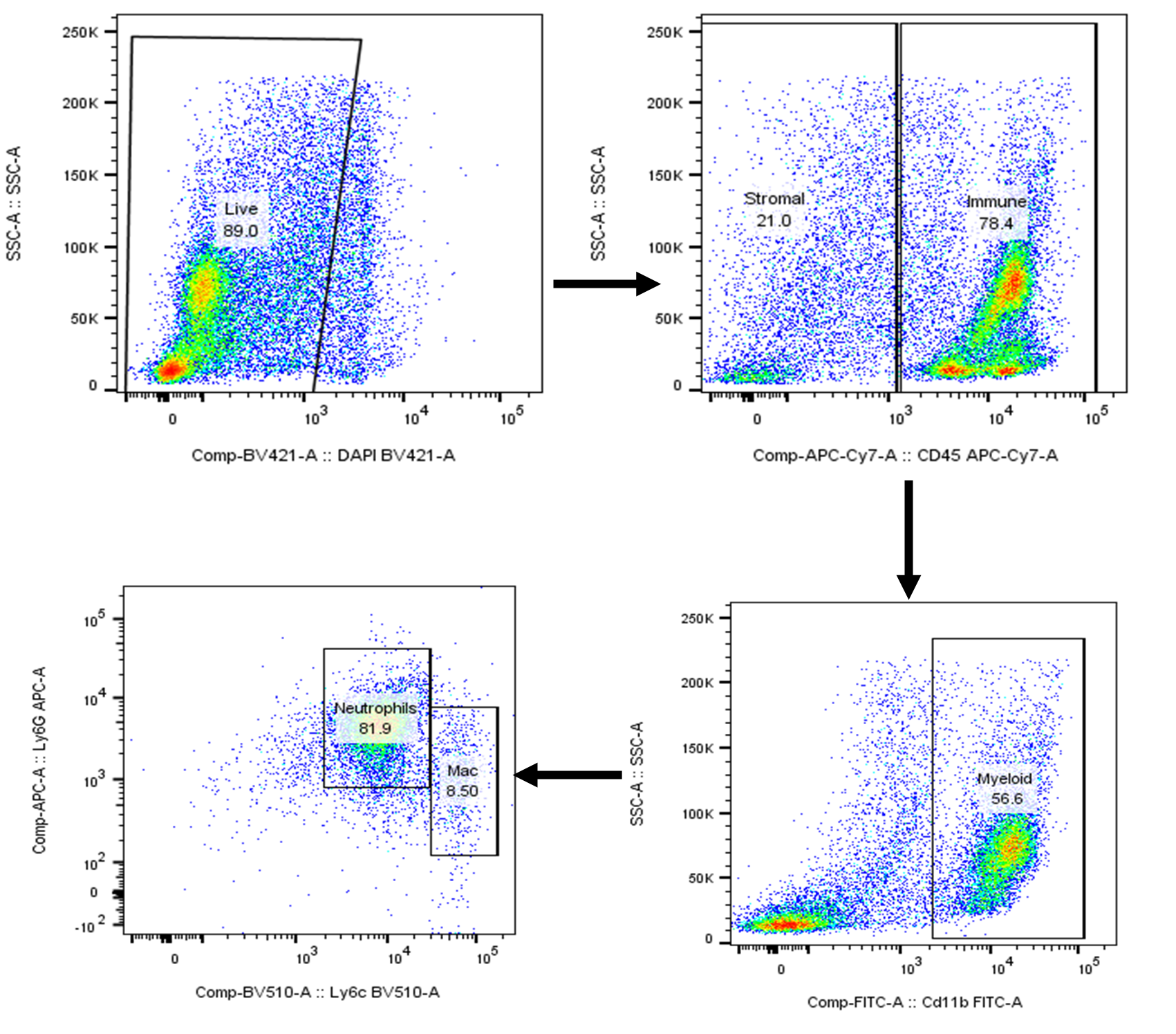


***Figure S2****. Flow cytometry gating strategy for Ly6c+ monocytes/macrophages (Mac).*


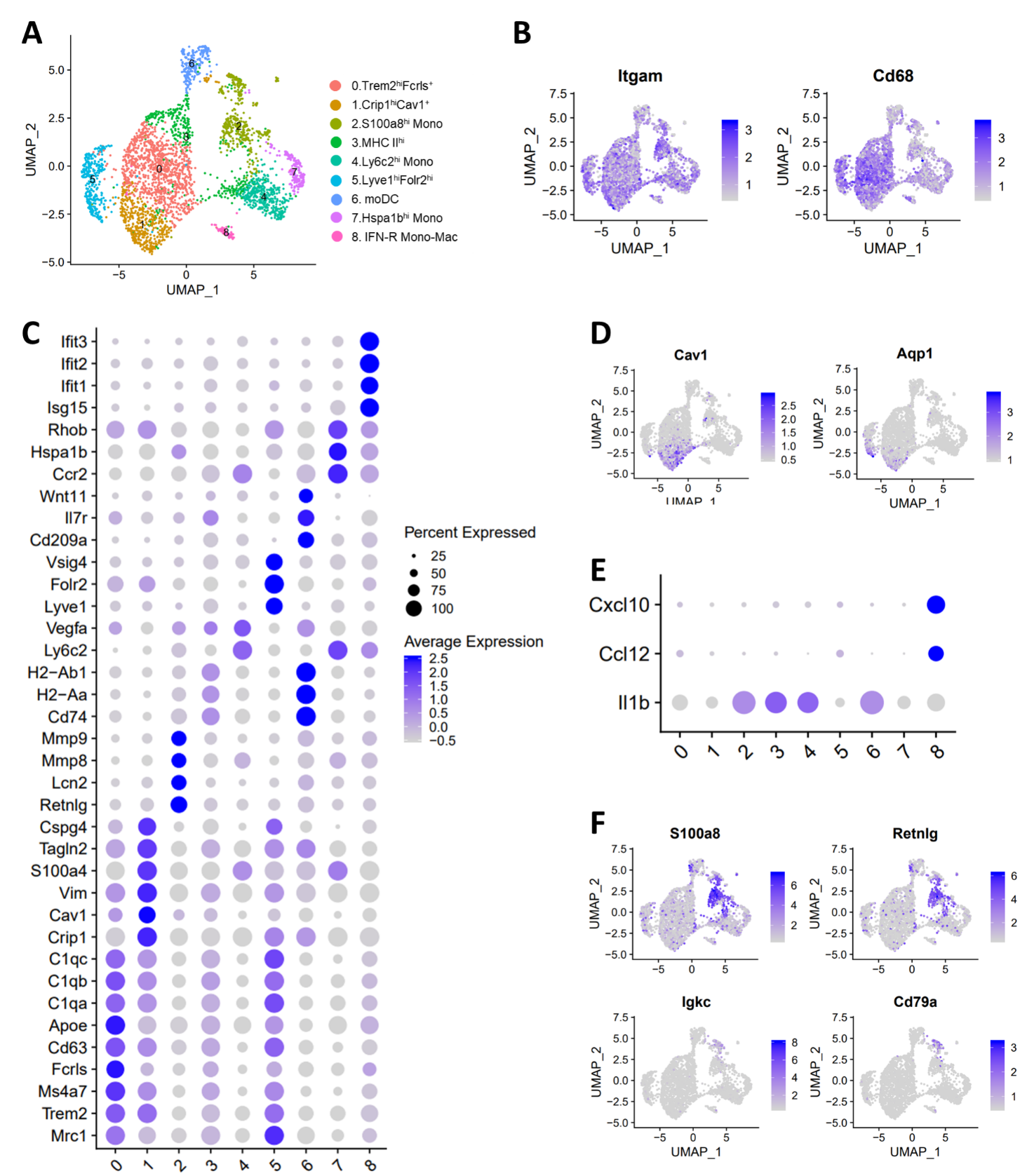


***Figure S3.*** *Characterization of monocyte and macrophages. A) UMAP plot showing various monocyte/macrophage clusters. B) Feature plot showing the expression of monocyte and macrophage markers. C) Dot plot showing markers of various monocyte and macrophage subtypes. D)Feature plot showing the expression of Cav1 and Aqp1. E) Dot pot showing the expression of selected inflammatory cytokines. F) Selected genes enriched in S100a8^hi^ monocytes.*


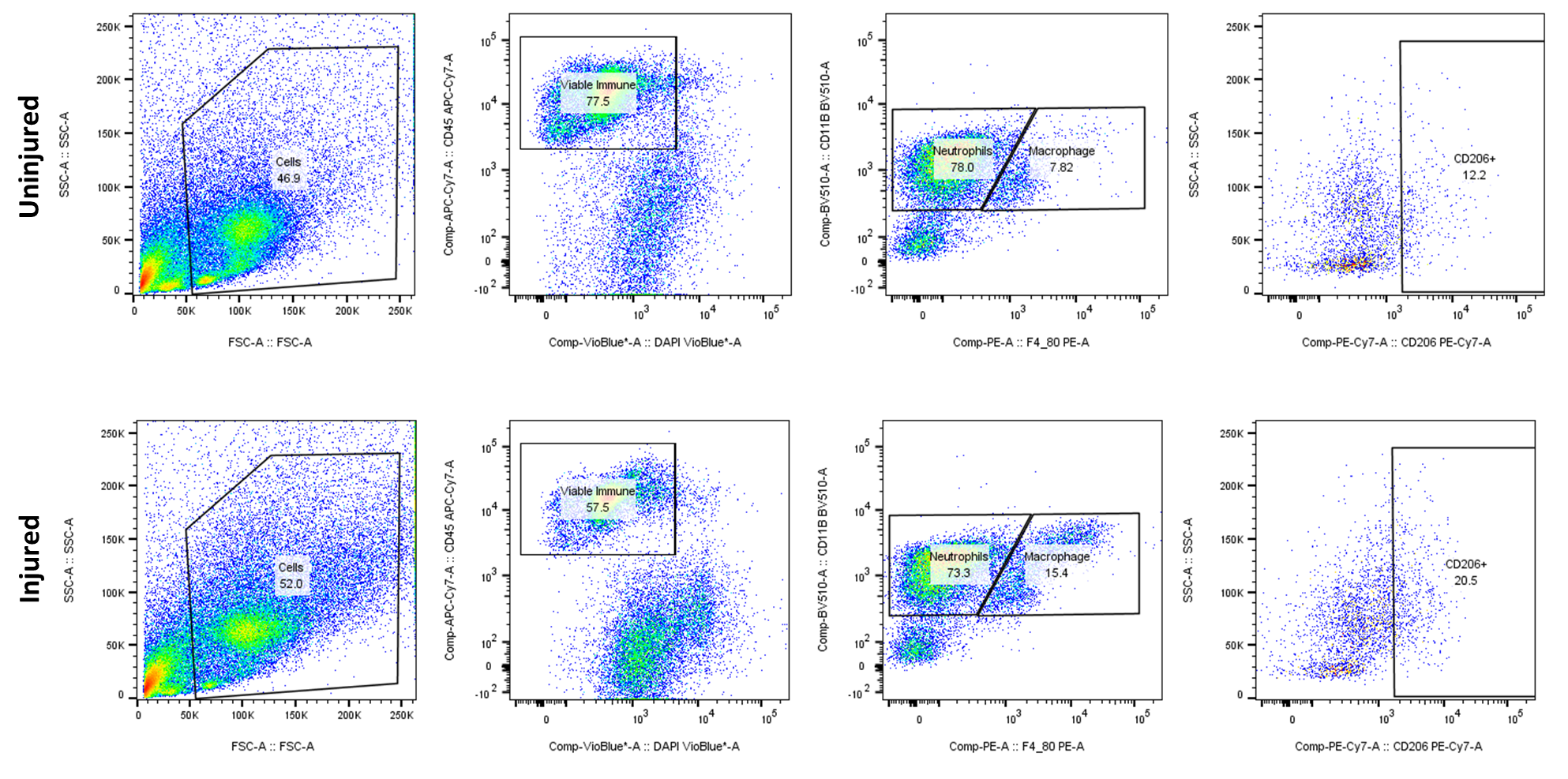


***Figure S4****. Flow cytometry gating strategy for M2-like macrophages. Immune cells from D0 (uninjured) and D7 (injured) were analyzed.*


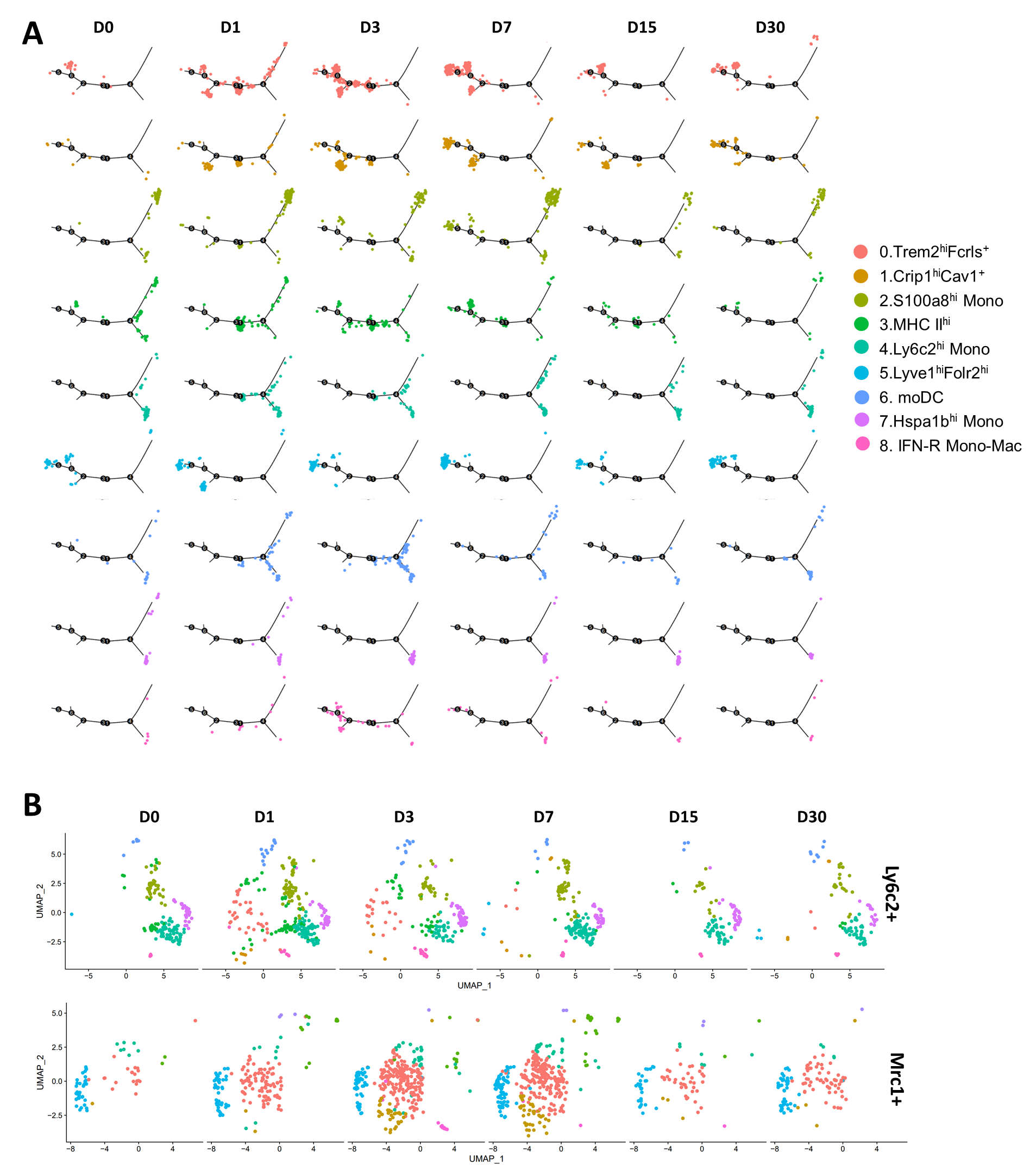


***Figure S5****. Pseudo-time differentiation trajectory analysis of monocyte/macrophages.* *A) Cells from each cluster at various timepoints mapped on the pseudo-time differentiation trajectory. Cells are colored based on clusters identities. B) UMAP plots showing only the Ly6c2+ and Mrc1+ cells (cells are colored based on clusters identities in panel A). A number of cells in Trem2^hi^Fcrls^+^ and MHC II^hi^ clusters were Ly6c2^+^ at D1 and D3 suggesting that these are monocyte-derived macrophages.*

**
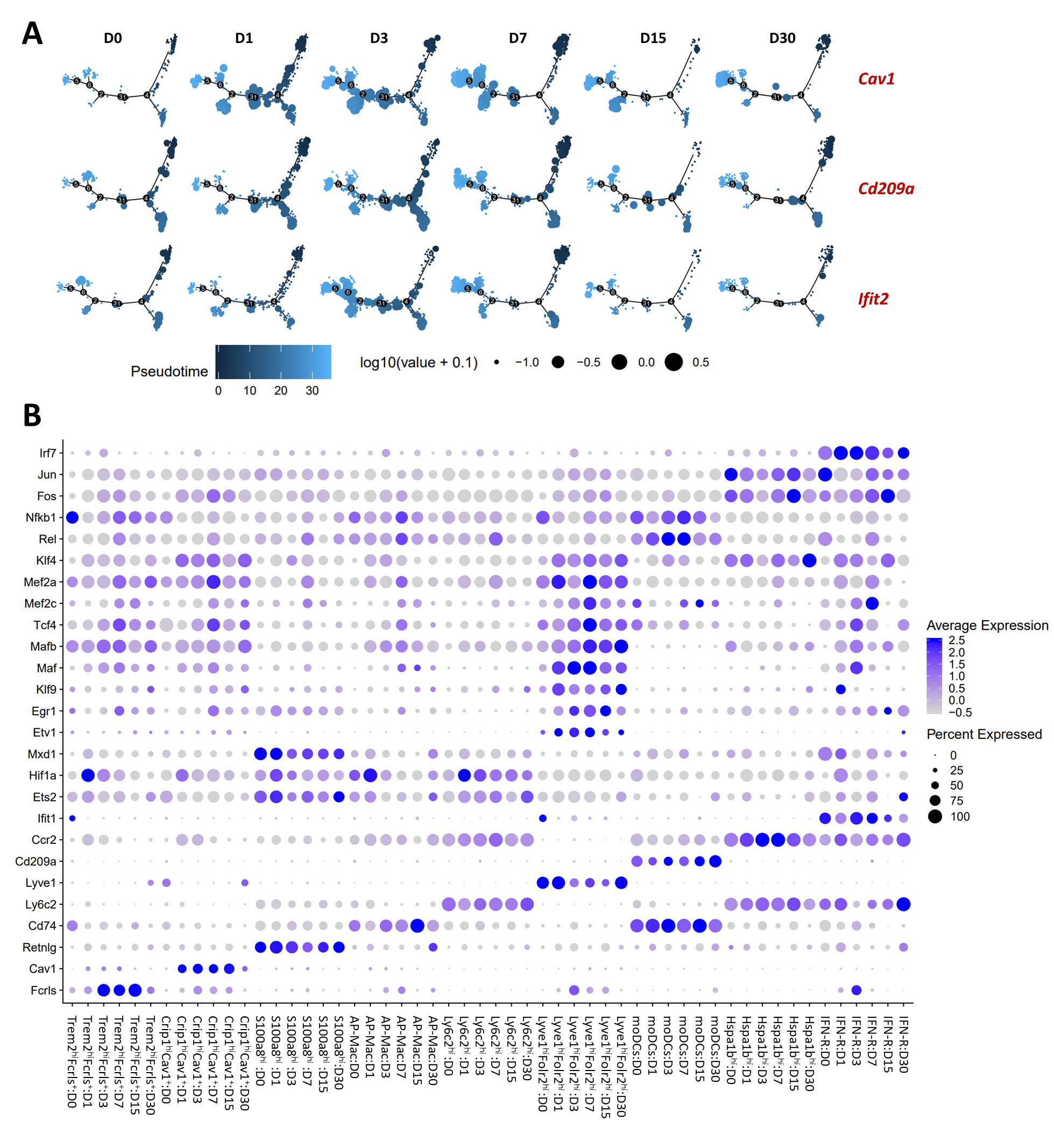
 Figure S6**. *A) Superimposition of the expression of selected genes on the pseudo-time trajectory (colored based on pseudo-time). Circle size represents the gene expression level. B) Dot plot showing transcription factors differentially expressed between various monocyte/macrophage clusters at D1-D30.*


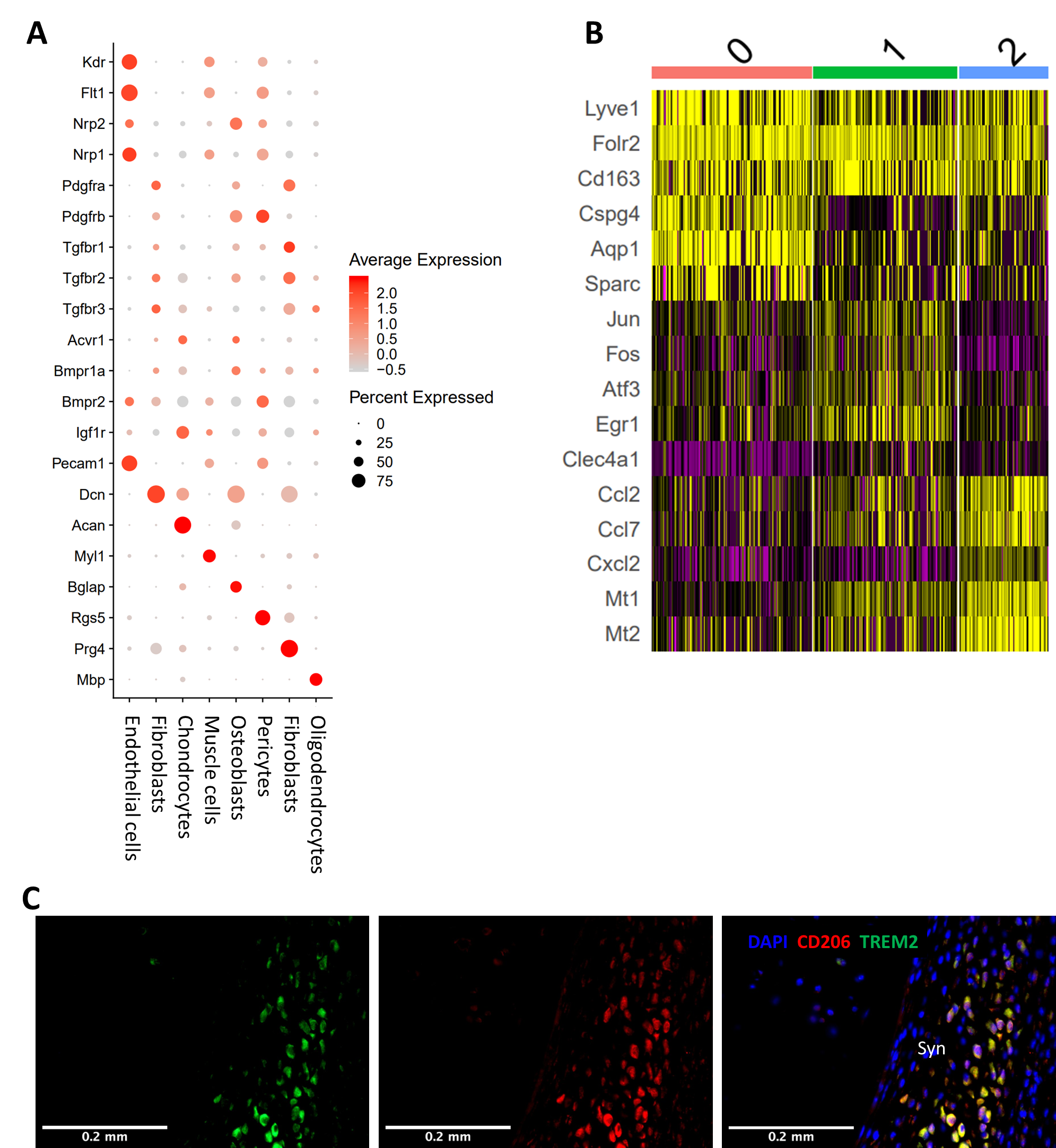


***Figure S7****. A) Dot plot showing the expression of markers of various non-immune cell clusters in the D0 joint along with growth factor receptors. B) Heatmap showing genes enriched in various Lyve1^hi^Folr2^hi^ macrophage subpopulations. C) Co-expression of CD206 and Trem2 in synovial macrophages at D7.*


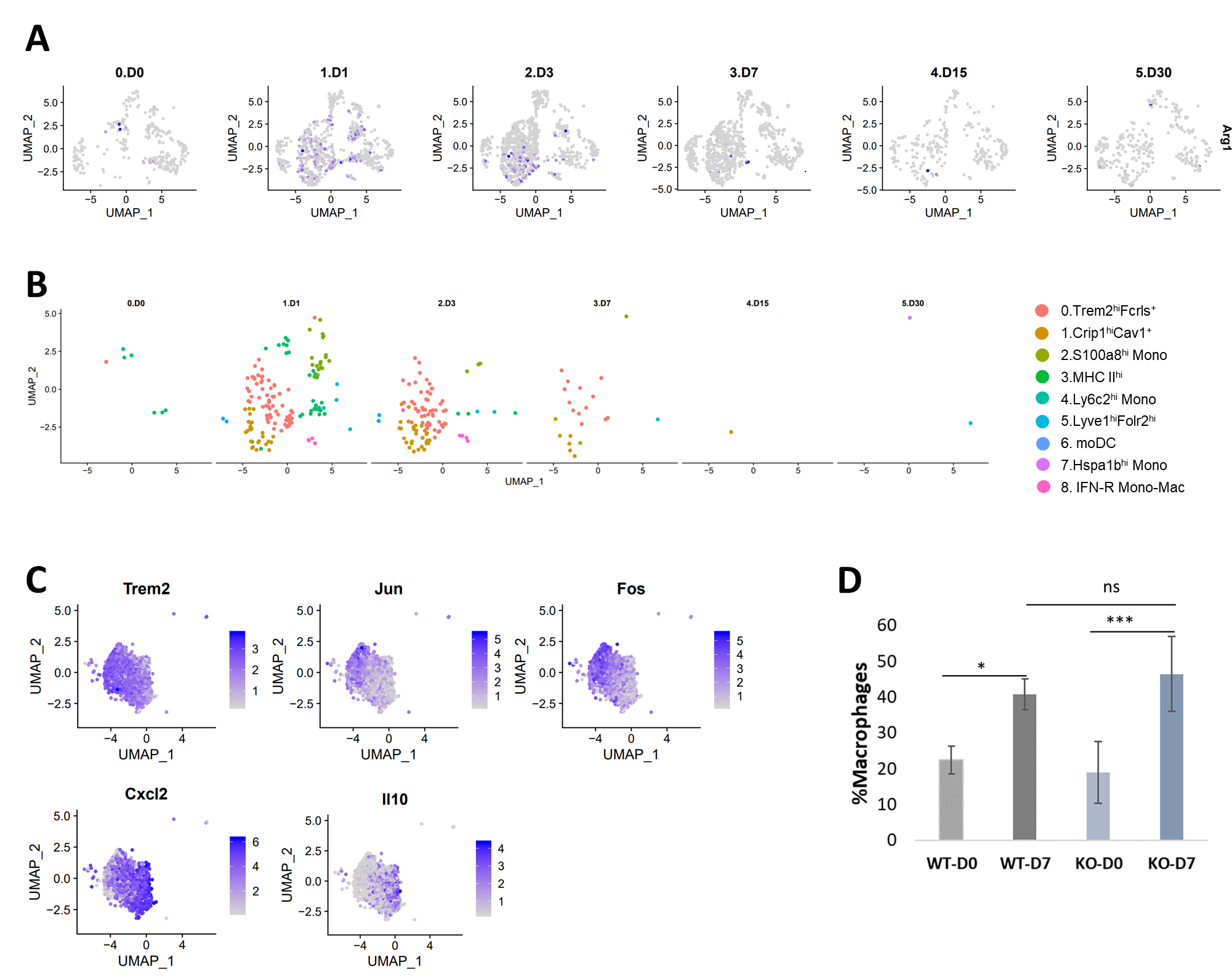


***Figure S8.*** *A) Feature plot showing Arg1 expression in monocytes/macrophages at various timepoints. B) UMAP plot of Arg1+ cells. Arg1 expression increased after injury but was significantly reduced by D7. C) Feature plots showing the expression of a subset of genes enriched in various Trem2+ infiltrating macrophage subtypes along with Trem2 expression. D) Proportion of M2-like macrophages in wildtype (WT) and Trem2^-/-^ (KO) mice at D0 and D7. * p ≤ 0.05; *** p ≤ 0.001; ns: not significant.*

**Table S1**: Markers of various Mono-Mac subclusters. Genes enriched in each cluster compared to all other clusters are shown in the table.

| **Gene** | **Cluster ID** | **Avg_logFC** | **P_val** | **P_val_adj** |
| --- | --- | --- | --- | --- |
| *Apoe* | 0 | 1.5898 | 3.60E-290 | 7.20E-287 |
| *Fcrls* | 0 | 1.1625 | 9.56E-232 | 1.91E-228 |
| *Ms4a7* | 0 | 1.1351 | 3.00E-278 | 6.01E-275 |
| *Cxcl1* | 0 | 1.0914 | 2.67E-88 | 5.34E-85 |
| *C1qb* | 0 | 0.9948 | 2.58E-197 | 5.17E-194 |
| *Cxcl2* | 0 | 0.9943 | 1.96E-111 | 3.92E-108 |
| *Il10* | 0 | 0.9690 | 2.13E-78 | 4.25E-75 |
| *Selenop* | 0 | 0.9449 | 1.53E-205 | 3.06E-202 |
| *Ctsb* | 0 | 0.8786 | 6.99E-248 | 1.40E-244 |
| *C1qa* | 0 | 0.8746 | 6.13E-161 | 1.23E-157 |
| *Abca1* | 0 | 0.8698 | 4.53E-233 | 9.07E-230 |
| *Spp1* | 0 | 0.8582 | 7.13E-111 | 1.43E-107 |
| *C1qc* | 0 | 0.8377 | 6.76E-168 | 1.35E-164 |
| *Cd63* | 0 | 0.8057 | 6.08E-209 | 1.22E-205 |
| *Gpnmb* | 0 | 0.8050 | 2.44E-122 | 4.88E-119 |
| *Fabp5* | 0 | 0.8046 | 4.30E-57 | 8.60E-54 |
| *Ctsd* | 0 | 0.8018 | 1.51E-183 | 3.02E-180 |
| *Gdf15* | 0 | 0.7939 | 2.76E-94 | 5.53E-91 |
| *Sdc4* | 0 | 0.7588 | 7.01E-164 | 1.40E-160 |
| *Trem2* | 0 | 0.7575 | 2.80E-185 | 5.59E-182 |
| *Lgmn* | 0 | 0.7519 | 3.37E-214 | 6.74E-211 |
| *Mrc1* | 0 | 0.7030 | 5.04E-157 | 1.01E-153 |
| *Pf4* | 0 | 0.7017 | 1.63E-125 | 3.26E-122 |
| *Clec4n* | 0 | 0.6946 | 2.73E-135 | 5.46E-132 |
| *Fnip2* | 0 | 0.6711 | 2.11E-109 | 4.22E-106 |
| *Adgre1* | 0 | 0.6695 | 2.88E-194 | 5.75E-191 |
| *Mmp14* | 0 | 0.6692 | 6.55E-169 | 1.31E-165 |
| *Sash1* | 0 | 0.6690 | 7.33E-195 | 1.47E-191 |
| *C3ar1* | 0 | 0.6677 | 4.35E-162 | 8.69E-159 |
| *Ctsl* | 0 | 0.6450 | 1.82E-141 | 3.63E-138 |
| *Ccl7* | 0 | 0.6402 | 3.83E-35 | 7.65E-32 |
| *Hmox1* | 0 | 0.6325 | 6.21E-66 | 1.24E-62 |
| *Pdpn* | 0 | 0.6290 | 2.78E-92 | 5.55E-89 |
| *Sqstm1* | 0 | 0.6227 | 6.23E-122 | 1.25E-118 |
| *Igf1* | 0 | 0.6190 | 6.58E-172 | 1.32E-168 |
| *Lmna* | 0 | 0.5986 | 8.10E-113 | 1.62E-109 |
| *Srxn1* | 0 | 0.5964 | 2.97E-93 | 5.95E-90 |
| *Dab2* | 0 | 0.5760 | 4.41E-129 | 8.82E-126 |
| *Rab7b* | 0 | 0.5741 | 5.12E-132 | 1.02E-128 |
| *Cxcl3* | 0 | 0.5706 | 1.65E-06 | 0.003305 |
| *Syngr1* | 0 | 0.5705 | 1.65E-129 | 3.29E-126 |
| *Ifi207* | 0 | 0.5680 | 2.63E-110 | 5.27E-107 |
| *Ms4a6d* | 0 | 0.5671 | 6.60E-141 | 1.32E-137 |
| *Fosb* | 0 | 0.5624 | 2.90E-52 | 5.79E-49 |
| *Lhfpl2* | 0 | 0.5507 | 2.24E-110 | 4.47E-107 |
| *Tnf* | 0 | 0.5419 | 2.89E-76 | 5.78E-73 |
| *Prdx1* | 0 | 0.5412 | 1.75E-58 | 3.50E-55 |
| *Il1rn* | 0 | 0.5374 | 2.16E-41 | 4.32E-38 |
| *Stab1* | 0 | 0.5369 | 2.08E-136 | 4.15E-133 |
| *Nrp2* | 0 | 0.5362 | 4.70E-119 | 9.41E-116 |
| *Pltp* | 0 | 0.5239 | 9.29E-112 | 1.86E-108 |
| *Errfi1* | 0 | 0.5188 | 4.46E-31 | 8.93E-28 |
| *Cxcl16* | 0 | 0.5162 | 2.97E-131 | 5.95E-128 |
| *Pdgfa* | 0 | 0.5112 | 6.01E-80 | 1.20E-76 |
| *Aif1* | 0 | 0.5089 | 1.15E-129 | 2.31E-126 |
| *Hpgds* | 0 | 0.5081 | 1.64E-143 | 3.29E-140 |
| *Itgav* | 0 | 0.5025 | 6.27E-106 | 1.25E-102 |
| *Tppp3* | 1 | 1.4292 | 5.40E-155 | 1.08E-151 |
| *Crip1* | 1 | 1.0306 | 4.59E-138 | 9.17E-135 |
| *Ecm1* | 1 | 0.9900 | 3.53E-121 | 7.06E-118 |
| *Vim* | 1 | 0.9320 | 1.10E-159 | 2.21E-156 |
| *S100a4* | 1 | 0.8615 | 9.08E-142 | 1.82E-138 |
| *Cspg4* | 1 | 0.8407 | 1.07E-137 | 2.13E-134 |
| *Cav1* | 1 | 0.8002 | 1.32E-107 | 2.64E-104 |
| *Aqp1* | 1 | 0.7803 | 1.28E-96 | 2.56E-93 |
| *Spp1* | 1 | 0.7488 | 9.20E-45 | 1.84E-41 |
| *S100a10* | 1 | 0.7270 | 9.11E-113 | 1.82E-109 |
| *Lgals1* | 1 | 0.7198 | 2.98E-124 | 5.96E-121 |
| *Ckb* | 1 | 0.7137 | 3.36E-97 | 6.72E-94 |
| *Pmepa1* | 1 | 0.7096 | 1.24E-92 | 2.49E-89 |
| *Tagln2* | 1 | 0.6972 | 8.25E-128 | 1.65E-124 |
| *Lmna* | 1 | 0.6839 | 2.55E-92 | 5.11E-89 |
| *Fn1* | 1 | 0.6674 | 3.38E-56 | 6.77E-53 |
| *Nfic* | 1 | 0.6114 | 3.69E-96 | 7.37E-93 |
| *Plec* | 1 | 0.6094 | 3.54E-92 | 7.07E-89 |
| *Aopep* | 1 | 0.5919 | 3.88E-85 | 7.77E-82 |
| *Olfml3* | 1 | 0.5889 | 9.53E-81 | 1.91E-77 |
| *Tubb5* | 1 | 0.5681 | 1.45E-85 | 2.90E-82 |
| *Pdlim1* | 1 | 0.5642 | 1.30E-90 | 2.60E-87 |
| *Rgcc* | 1 | 0.5628 | 4.20E-47 | 8.39E-44 |
| *Myof* | 1 | 0.5617 | 1.58E-93 | 3.15E-90 |
| *Cyb5r3* | 1 | 0.5600 | 6.68E-86 | 1.34E-82 |
| *Klf4* | 1 | 0.5527 | 2.74E-50 | 5.48E-47 |
| *Ahnak* | 1 | 0.5493 | 9.14E-86 | 1.83E-82 |
| *Anxa1* | 1 | 0.5415 | 2.94E-85 | 5.87E-82 |
| *Lrp1* | 1 | 0.5300 | 3.43E-81 | 6.86E-78 |
| *Capn2* | 1 | 0.5287 | 1.48E-93 | 2.96E-90 |
| *Pltp* | 1 | 0.5205 | 1.02E-48 | 2.04E-45 |
| *Mgst3* | 1 | 0.5155 | 9.91E-94 | 1.98E-90 |
| *Lair1* | 1 | 0.5140 | 2.68E-75 | 5.35E-72 |
| *Pf4* | 1 | 0.5066 | 1.53E-42 | 3.05E-39 |
| *Emp1* | 1 | 0.5053 | 4.61E-65 | 9.23E-62 |
| *Retnlg* | 2 | 2.8756 | 7.42E-102 | 1.48E-98 |
| *Ngp* | 2 | 2.8558 | 7.64E-44 | 1.53E-40 |
| *Camp* | 2 | 2.7155 | 4.67E-45 | 9.33E-42 |
| *Igkc* | 2 | 2.5399 | 2.57E-09 | 5.14E-06 |
| *Ltf* | 2 | 2.2572 | 3.91E-54 | 7.82E-51 |
| *Lcn2* | 2 | 2.2317 | 3.03E-90 | 6.06E-87 |
| *G0s2* | 2 | 1.9554 | 2.52E-102 | 5.04E-99 |
| *Acod1* | 2 | 1.7638 | 2.95E-67 | 5.91E-64 |
| *Wfdc21* | 2 | 1.6487 | 3.93E-60 | 7.85E-57 |
| *Mmp8* | 2 | 1.5654 | 2.34E-59 | 4.68E-56 |
| *Mmp9* | 2 | 1.4332 | 7.63E-86 | 1.53E-82 |
| *Ighm* | 2 | 1.0473 | 2.38E-29 | 4.76E-26 |
| *Cstdc4* | 2 | 1.0456 | 8.19E-10 | 1.64E-06 |
| *Cd24a* | 2 | 0.9205 | 5.72E-76 | 1.14E-72 |
| *Ifitm1* | 2 | 0.9082 | 9.23E-22 | 1.85E-18 |
| *Ly6d* | 2 | 0.8546 | 1.96E-11 | 3.92E-08 |
| *Ifitm6* | 2 | 0.8486 | 5.03E-45 | 1.01E-41 |
| *Hcar2* | 2 | 0.7970 | 1.76E-35 | 3.51E-32 |
| *Syne1* | 2 | 0.7766 | 2.68E-27 | 5.36E-24 |
| *Stfa2l1* | 2 | 0.7449 | 4.99E-13 | 9.98E-10 |
| *Asprv1* | 2 | 0.7210 | 3.25E-06 | 0.006498 |
| *Ebf1* | 2 | 0.7109 | 2.56E-06 | 0.005129 |
| *Ly6g* | 2 | 0.7095 | 9.50E-34 | 1.90E-30 |
| *Adpgk* | 2 | 0.7044 | 6.80E-33 | 1.36E-29 |
| *Gadd45a* | 2 | 0.7001 | 3.66E-29 | 7.33E-26 |
| *Cd177* | 2 | 0.6950 | 4.62E-34 | 9.24E-31 |
| *Hmgb2* | 2 | 0.6946 | 9.01E-55 | 1.80E-51 |
| *Fabp4* | 2 | 0.6823 | 5.73E-15 | 1.15E-11 |
| *Rgs2* | 2 | 0.6321 | 3.78E-29 | 7.57E-26 |
| *Chil3* | 2 | 0.6116 | 6.38E-15 | 1.28E-11 |
| *Ace* | 2 | 0.5958 | 1.36E-24 | 2.72E-21 |
| *Chil1* | 2 | 0.5846 | 5.94E-35 | 1.19E-31 |
| *Krt83* | 2 | 0.5636 | 2.05E-23 | 4.10E-20 |
| *Cks2* | 2 | 0.5632 | 1.69E-08 | 3.38E-05 |
| *Adgre4* | 2 | 0.5580 | 5.32E-46 | 1.06E-42 |
| *Cd79b* | 2 | 0.5580 | 2.15E-20 | 4.30E-17 |
| *Rflnb* | 2 | 0.5512 | 1.48E-38 | 2.95E-35 |
| *Il1f9* | 2 | 0.5317 | 1.57E-16 | 3.14E-13 |
| *Satb1* | 2 | 0.5212 | 9.84E-39 | 1.97E-35 |
| *Il1b* | 2 | 0.5202 | 8.73E-33 | 1.75E-29 |
| *Olfm4* | 2 | 0.5090 | 1.57E-17 | 3.14E-14 |
| *Vpreb3* | 2 | 0.5075 | 6.50E-06 | 0.012999 |
| *H2-Eb1* | 3 | 1.1622 | 2.78E-53 | 5.56E-50 |
| *Cd74* | 3 | 1.1401 | 2.59E-63 | 5.18E-60 |
| *H2-Ab1* | 3 | 1.1326 | 1.27E-70 | 2.53E-67 |
| *H2-Aa* | 3 | 1.0698 | 1.24E-60 | 2.49E-57 |
| *Tnip3* | 3 | 0.6678 | 7.08E-54 | 1.42E-50 |
| *Tmem176a* | 3 | 0.5995 | 2.64E-31 | 5.27E-28 |
| *Tmem176b* | 3 | 0.5167 | 2.61E-23 | 5.22E-20 |
| *Plac8* | 4 | 1.5612 | 2.17E-153 | 4.35E-150 |
| *Ly6c2* | 4 | 1.4631 | 1.44E-154 | 2.87E-151 |
| *Tmsb10* | 4 | 1.4411 | 1.97E-162 | 3.94E-159 |
| *Vcan* | 4 | 1.3082 | 2.44E-118 | 4.88E-115 |
| *Chil3* | 4 | 1.2836 | 1.43E-99 | 2.86E-96 |
| *C3* | 4 | 1.1699 | 2.31E-152 | 4.61E-149 |
| *F10* | 4 | 1.1644 | 4.06E-134 | 8.11E-131 |
| *Ifitm6* | 4 | 1.1091 | 4.74E-140 | 9.47E-137 |
| *Ccr2* | 4 | 0.9294 | 1.48E-115 | 2.97E-112 |
| *Fn1* | 4 | 0.9188 | 1.55E-96 | 3.10E-93 |
| *Nr4a2* | 4 | 0.8338 | 4.70E-84 | 9.40E-81 |
| *Nrg1* | 4 | 0.7986 | 1.54E-98 | 3.08E-95 |
| *Itgb7* | 4 | 0.7801 | 2.52E-109 | 5.03E-106 |
| *Plcb1* | 4 | 0.7421 | 1.60E-136 | 3.20E-133 |
| *Rplp0* | 4 | 0.7098 | 3.09E-153 | 6.18E-150 |
| *Rpsa* | 4 | 0.7016 | 6.53E-131 | 1.31E-127 |
| *Ms4a4c* | 4 | 0.6785 | 2.31E-94 | 4.62E-91 |
| *Adora2b* | 4 | 0.6779 | 3.38E-89 | 6.76E-86 |
| *Ccl9* | 4 | 0.6426 | 3.25E-80 | 6.50E-77 |
| *Hmgb2* | 4 | 0.6137 | 4.82E-67 | 9.64E-64 |
| *Rps6* | 4 | 0.6137 | 7.04E-122 | 1.41E-118 |
| *Uck2* | 4 | 0.6133 | 5.35E-82 | 1.07E-78 |
| *F13a1* | 4 | 0.5840 | 1.38E-64 | 2.77E-61 |
| *Ms4a6c* | 4 | 0.5689 | 2.09E-83 | 4.18E-80 |
| *Il1b* | 4 | 0.5388 | 1.30E-19 | 2.61E-16 |
| *Apoc2* | 4 | 0.5318 | 2.43E-56 | 4.86E-53 |
| *Rps5* | 4 | 0.5137 | 3.55E-110 | 7.10E-107 |
| *Rack1* | 4 | 0.5091 | 8.07E-109 | 1.61E-105 |
| *Ldlr* | 4 | 0.5067 | 1.44E-58 | 2.89E-55 |
| *Vsig4* | 5 | 2.3092 | 1.41E-122 | 2.82E-119 |
| *Lyve1* | 5 | 1.7114 | 6.37E-125 | 1.27E-121 |
| *Cfh* | 5 | 1.4598 | 3.33E-167 | 6.67E-164 |
| *Folr2* | 5 | 1.4195 | 5.50E-160 | 1.10E-156 |
| *Gas6* | 5 | 1.4038 | 2.88E-162 | 5.75E-159 |
| *Cd163* | 5 | 1.3753 | 4.07E-125 | 8.14E-122 |
| *Timp2* | 5 | 1.2787 | 1.35E-159 | 2.71E-156 |
| *Cd36* | 5 | 1.1322 | 1.16E-112 | 2.33E-109 |
| *Cbr2* | 5 | 1.1181 | 2.06E-115 | 4.12E-112 |
| *Aqp1* | 5 | 1.1058 | 2.07E-66 | 4.14E-63 |
| *Serpinb6a* | 5 | 1.0846 | 1.70E-140 | 3.39E-137 |
| *Fxyd2* | 5 | 1.0842 | 1.98E-116 | 3.95E-113 |
| *Colec12* | 5 | 1.0797 | 1.21E-155 | 2.41E-152 |
| *Tns1* | 5 | 1.0590 | 1.30E-140 | 2.60E-137 |
| *Rnase4* | 5 | 1.0561 | 6.60E-131 | 1.32E-127 |
| *Pmp22* | 5 | 1.0430 | 1.71E-137 | 3.41E-134 |
| *Fcgrt* | 5 | 1.0160 | 3.14E-140 | 6.29E-137 |
| *Mrc1* | 5 | 0.9836 | 1.25E-92 | 2.50E-89 |
| *F13a1* | 5 | 0.9763 | 1.44E-104 | 2.88E-101 |
| *Maf* | 5 | 0.9658 | 2.00E-113 | 4.00E-110 |
| *Fgfr1* | 5 | 0.9576 | 6.62E-150 | 1.32E-146 |
| *Selenop* | 5 | 0.9447 | 5.90E-82 | 1.18E-78 |
| *Hmox1* | 5 | 0.9290 | 1.82E-58 | 3.64E-55 |
| *C4b* | 5 | 0.9255 | 7.81E-129 | 1.56E-125 |
| *Ednrb* | 5 | 0.9117 | 7.31E-55 | 1.46E-51 |
| *Ctla2a* | 5 | 0.8838 | 6.51E-87 | 1.30E-83 |
| *Stab1* | 5 | 0.8781 | 5.55E-103 | 1.11E-99 |
| *Hpgd* | 5 | 0.8639 | 1.44E-117 | 2.87E-114 |
| *Sulf2* | 5 | 0.8586 | 1.14E-122 | 2.28E-119 |
| *Dab2* | 5 | 0.8472 | 6.57E-103 | 1.31E-99 |
| *Pmepa1* | 5 | 0.8429 | 8.71E-67 | 1.74E-63 |
| *Ctsd* | 5 | 0.8346 | 3.39E-68 | 6.77E-65 |
| *C1qc* | 5 | 0.8317 | 9.47E-90 | 1.89E-86 |
| *Emp1* | 5 | 0.8178 | 6.51E-86 | 1.30E-82 |
| *Ifi27l2a* | 5 | 0.8059 | 6.83E-67 | 1.37E-63 |
| *Igf1* | 5 | 0.8043 | 1.21E-95 | 2.43E-92 |
| *Cd81* | 5 | 0.7983 | 5.53E-112 | 1.11E-108 |
| *C1qa* | 5 | 0.7780 | 1.69E-81 | 3.37E-78 |
| *Wwp1* | 5 | 0.7753 | 8.06E-109 | 1.61E-105 |
| *Grn* | 5 | 0.7580 | 4.66E-114 | 9.32E-111 |
| *Pf4* | 5 | 0.7498 | 1.41E-69 | 2.81E-66 |
| *Ecm1* | 5 | 0.7478 | 1.97E-81 | 3.95E-78 |
| *Camk1* | 5 | 0.7455 | 1.73E-101 | 3.45E-98 |
| *Wfdc17* | 5 | 0.7426 | 1.62E-42 | 3.25E-39 |
| *Ccl7* | 5 | 0.7374 | 2.51E-32 | 5.02E-29 |
| *Gpr34* | 5 | 0.7358 | 8.54E-81 | 1.71E-77 |
| *Sparc* | 5 | 0.7357 | 3.36E-28 | 6.71E-25 |
| *Mafb* | 5 | 0.7272 | 2.30E-67 | 4.60E-64 |
| *Nupr1* | 5 | 0.7098 | 3.42E-62 | 6.84E-59 |
| *Pros1* | 5 | 0.7055 | 1.49E-111 | 2.98E-108 |
| *Mt1* | 5 | 0.6857 | 2.23E-40 | 4.45E-37 |
| *Hexb* | 5 | 0.6849 | 1.31E-66 | 2.63E-63 |
| *Ltc4s* | 5 | 0.6818 | 4.76E-70 | 9.52E-67 |
| *Ccl2* | 5 | 0.6759 | 1.87E-27 | 3.73E-24 |
| *Lgmn* | 5 | 0.6721 | 2.49E-77 | 4.99E-74 |
| *Ccl8* | 5 | 0.6710 | 1.07E-28 | 2.14E-25 |
| *Mt2* | 5 | 0.6670 | 1.49E-34 | 2.97E-31 |
| *Tcim* | 5 | 0.6659 | 7.09E-106 | 1.42E-102 |
| *Pepd* | 5 | 0.6638 | 1.23E-98 | 2.46E-95 |
| *Csf1r* | 5 | 0.6559 | 1.20E-102 | 2.39E-99 |
| *Pltp* | 5 | 0.6531 | 3.58E-75 | 7.15E-72 |
| *Mtmr10* | 5 | 0.6483 | 1.17E-70 | 2.33E-67 |
| *Hpgds* | 5 | 0.6378 | 8.19E-86 | 1.64E-82 |
| *Scoc* | 5 | 0.6283 | 6.32E-94 | 1.26E-90 |
| *Sptbn1* | 5 | 0.6204 | 2.30E-84 | 4.59E-81 |
| *Lima1* | 5 | 0.6130 | 4.35E-87 | 8.70E-84 |
| *Nrp2* | 5 | 0.6022 | 2.71E-75 | 5.42E-72 |
| *Arsb* | 5 | 0.6018 | 4.89E-67 | 9.77E-64 |
| *Bin1* | 5 | 0.5975 | 2.32E-83 | 4.64E-80 |
| *Glul* | 5 | 0.5940 | 1.01E-70 | 2.01E-67 |
| *Ctla2b* | 5 | 0.5874 | 1.87E-79 | 3.75E-76 |
| *Nrp1* | 5 | 0.5789 | 1.63E-80 | 3.26E-77 |
| *Blvrb* | 5 | 0.5762 | 1.56E-73 | 3.13E-70 |
| *Dpysl3* | 5 | 0.5665 | 2.49E-112 | 4.99E-109 |
| *Slco2b1* | 5 | 0.5623 | 4.94E-81 | 9.87E-78 |
| *Cspg4* | 5 | 0.5574 | 1.76E-42 | 3.52E-39 |
| *Ccn1* | 5 | 0.5474 | 6.69E-70 | 1.34E-66 |
| *Ctsb* | 5 | 0.5410 | 1.48E-59 | 2.95E-56 |
| *Spire1* | 5 | 0.5408 | 1.60E-86 | 3.20E-83 |
| *Stard8* | 5 | 0.5398 | 4.95E-75 | 9.90E-72 |
| *Tppp3* | 5 | 0.5378 | 1.11E-48 | 2.23E-45 |
| *Egr1* | 5 | 0.5374 | 9.15E-32 | 1.83E-28 |
| *Ehd4* | 5 | 0.5259 | 1.29E-65 | 2.57E-62 |
| *Rgs10* | 5 | 0.5242 | 2.14E-68 | 4.29E-65 |
| *Tcf4* | 5 | 0.5241 | 1.02E-67 | 2.03E-64 |
| *S1pr1* | 5 | 0.5221 | 3.99E-91 | 7.98E-88 |
| *C3ar1* | 5 | 0.5215 | 6.10E-55 | 1.22E-51 |
| *Rcan1* | 5 | 0.5181 | 4.77E-31 | 9.55E-28 |
| *Nfic* | 5 | 0.5177 | 5.13E-55 | 1.03E-51 |
| *Npl* | 5 | 0.5175 | 8.77E-79 | 1.75E-75 |
| *Tuba1a* | 5 | 0.5168 | 2.75E-54 | 5.49E-51 |
| *Zbtb20* | 5 | 0.5163 | 1.14E-60 | 2.28E-57 |
| *Akr1b8* | 5 | 0.5151 | 8.59E-79 | 1.72E-75 |
| *Rasgrp3* | 5 | 0.5046 | 7.26E-91 | 1.45E-87 |
| *Bmp2* | 5 | 0.5015 | 5.24E-61 | 1.05E-57 |
| *Dst* | 5 | 0.5013 | 1.56E-78 | 3.11E-75 |
| *H2-Ab1* | 6 | 2.4479 | 1.18E-126 | 2.37E-123 |
| *H2-Eb1* | 6 | 2.4277 | 1.29E-125 | 2.57E-122 |
| *H2-Aa* | 6 | 2.4162 | 1.11E-125 | 2.23E-122 |
| *Cd74* | 6 | 2.3567 | 2.80E-125 | 5.59E-122 |
| *Cd209a* | 6 | 1.6864 | 1.63E-71 | 3.27E-68 |
| *Tnip3* | 6 | 1.3404 | 3.22E-58 | 6.45E-55 |
| *H2-DMb1* | 6 | 1.2667 | 8.00E-105 | 1.60E-101 |
| *Il1b* | 6 | 1.1907 | 5.79E-47 | 1.16E-43 |
| *H2-DMa* | 6 | 1.1864 | 1.41E-106 | 2.83E-103 |
| *Malt1* | 6 | 1.1349 | 1.43E-65 | 2.86E-62 |
| *Etv3* | 6 | 1.1124 | 6.74E-70 | 1.35E-66 |
| *Mgl2* | 6 | 1.0947 | 1.55E-96 | 3.11E-93 |
| *Ccr7* | 6 | 1.0861 | 2.39E-09 | 4.79E-06 |
| *Klrb1b* | 6 | 1.0623 | 4.39E-96 | 8.79E-93 |
| *Klrd1* | 6 | 1.0372 | 2.69E-60 | 5.38E-57 |
| *Nr4a3* | 6 | 0.9876 | 3.67E-51 | 7.34E-48 |
| *Tbc1d4* | 6 | 0.9266 | 4.89E-26 | 9.78E-23 |
| *Il7r* | 6 | 0.8942 | 7.23E-37 | 1.45E-33 |
| *Klrk1* | 6 | 0.8932 | 4.66E-90 | 9.32E-87 |
| *P2ry10* | 6 | 0.8573 | 2.23E-78 | 4.46E-75 |
| *Grasp* | 6 | 0.8298 | 9.03E-72 | 1.81E-68 |
| *Flt3* | 6 | 0.7851 | 1.34E-97 | 2.68E-94 |
| *Ifitm1* | 6 | 0.7801 | 3.98E-11 | 7.96E-08 |
| *Traf1* | 6 | 0.7709 | 1.19E-49 | 2.38E-46 |
| *Runx3* | 6 | 0.7663 | 1.94E-58 | 3.89E-55 |
| *Ccl5* | 6 | 0.7593 | 2.67E-44 | 5.33E-41 |
| *H2afy* | 6 | 0.7489 | 1.56E-67 | 3.12E-64 |
| *Clec10a* | 6 | 0.7477 | 1.12E-32 | 2.24E-29 |
| *Plscr1* | 6 | 0.7431 | 2.37E-65 | 4.74E-62 |
| *Ccnd1* | 6 | 0.7370 | 1.11E-58 | 2.23E-55 |
| *Cd7* | 6 | 0.7317 | 1.02E-57 | 2.03E-54 |
| *Ramp3* | 6 | 0.7263 | 5.07E-31 | 1.01E-27 |
| *Wnt11* | 6 | 0.7132 | 2.14E-40 | 4.28E-37 |
| *Rpsa* | 6 | 0.7068 | 1.33E-79 | 2.66E-76 |
| *Cenpa* | 6 | 0.6950 | 1.72E-31 | 3.44E-28 |
| *Cst3* | 6 | 0.6856 | 9.85E-44 | 1.97E-40 |
| *Itgb7* | 6 | 0.6815 | 6.63E-61 | 1.33E-57 |
| *Gpr171* | 6 | 0.6767 | 5.13E-26 | 1.03E-22 |
| *Cd86* | 6 | 0.6673 | 3.33E-47 | 6.67E-44 |
| *Cbfa2t3* | 6 | 0.6638 | 2.29E-42 | 4.59E-39 |
| *Olfm1* | 6 | 0.6584 | 1.49E-58 | 2.98E-55 |
| *Rpl12* | 6 | 0.6508 | 1.59E-76 | 3.17E-73 |
| *Ifi30* | 6 | 0.6452 | 3.62E-54 | 7.24E-51 |
| *Fyn* | 6 | 0.6427 | 5.71E-44 | 1.14E-40 |
| *Ciita* | 6 | 0.6306 | 9.57E-81 | 1.91E-77 |
| *Bhlhe40* | 6 | 0.6249 | 2.83E-24 | 5.65E-21 |
| *H2afz* | 6 | 0.6015 | 3.15E-52 | 6.29E-49 |
| *Rpl14* | 6 | 0.5978 | 2.87E-78 | 5.74E-75 |
| *St8sia4* | 6 | 0.5846 | 4.51E-20 | 9.02E-17 |
| *Eef1b2* | 6 | 0.5723 | 1.86E-61 | 3.71E-58 |
| *Mpp6* | 6 | 0.5640 | 8.59E-57 | 1.72E-53 |
| *Tmem176a* | 6 | 0.5584 | 1.48E-27 | 2.96E-24 |
| *Gpr183* | 6 | 0.5527 | 2.00E-11 | 4.01E-08 |
| *Avpi1* | 6 | 0.5415 | 2.17E-38 | 4.33E-35 |
| *Flrt3* | 6 | 0.5406 | 6.60E-22 | 1.32E-18 |
| *Tmsb10* | 6 | 0.5385 | 2.80E-33 | 5.61E-30 |
| *Siglecg* | 6 | 0.5284 | 2.85E-17 | 5.71E-14 |
| *Ece1* | 6 | 0.5252 | 5.34E-71 | 1.07E-67 |
| *Ccnd2* | 6 | 0.5247 | 3.02E-47 | 6.04E-44 |
| *Pmaip1* | 6 | 0.5130 | 1.08E-24 | 2.15E-21 |
| *H2-Ob* | 6 | 0.5112 | 1.29E-72 | 2.57E-69 |
| *Rpl32* | 6 | 0.5056 | 6.49E-56 | 1.30E-52 |
| *Grk3* | 6 | 0.5014 | 4.12E-66 | 8.24E-63 |
| *Ly6c2* | 7 | 1.5352 | 3.30E-81 | 6.60E-78 |
| *Plac8* | 7 | 1.5157 | 2.31E-84 | 4.62E-81 |
| *Ccr2* | 7 | 1.4896 | 6.21E-91 | 1.24E-87 |
| *Tmsb10* | 7 | 1.4262 | 2.36E-84 | 4.73E-81 |
| *Hspa1b* | 7 | 1.4025 | 4.01E-49 | 8.02E-46 |
| *Chil3* | 7 | 1.3519 | 3.08E-44 | 6.17E-41 |
| *Hspa1a* | 7 | 1.1997 | 2.07E-47 | 4.13E-44 |
| *Rgs2* | 7 | 1.1093 | 4.33E-54 | 8.66E-51 |
| *Ifitm6* | 7 | 1.0135 | 9.03E-70 | 1.81E-66 |
| *Ms4a4c* | 7 | 0.9827 | 2.66E-67 | 5.32E-64 |
| *C3* | 7 | 0.9155 | 6.14E-66 | 1.23E-62 |
| *Apoc2* | 7 | 0.9013 | 1.77E-58 | 3.53E-55 |
| *Jun* | 7 | 0.8832 | 8.96E-39 | 1.79E-35 |
| *Ifi27l2a* | 7 | 0.8480 | 4.33E-38 | 8.67E-35 |
| *Tmpo* | 7 | 0.8394 | 4.64E-67 | 9.29E-64 |
| *Ms4a6c* | 7 | 0.8227 | 3.29E-71 | 6.58E-68 |
| *Fn1* | 7 | 0.7975 | 2.32E-45 | 4.64E-42 |
| *Itgb7* | 7 | 0.7716 | 2.99E-59 | 5.98E-56 |
| *Klf2* | 7 | 0.7235 | 7.98E-29 | 1.60E-25 |
| *Rhob* | 7 | 0.7141 | 1.76E-29 | 3.53E-26 |
| *Rpsa* | 7 | 0.7061 | 1.74E-70 | 3.49E-67 |
| *Rnase6* | 7 | 0.7000 | 2.13E-62 | 4.26E-59 |
| *Hmgb2* | 7 | 0.6960 | 1.65E-57 | 3.31E-54 |
| *Tent5a* | 7 | 0.6655 | 3.76E-39 | 7.52E-36 |
| *Clec4a3* | 7 | 0.6386 | 2.69E-46 | 5.39E-43 |
| *Pld4* | 7 | 0.6381 | 1.76E-59 | 3.52E-56 |
| *Ccl3* | 7 | 0.6248 | 2.71E-17 | 5.43E-14 |
| *F10* | 7 | 0.5919 | 2.06E-43 | 4.11E-40 |
| *Wfdc17* | 7 | 0.5722 | 1.99E-29 | 3.98E-26 |
| *Rps5* | 7 | 0.5694 | 8.85E-69 | 1.77E-65 |
| *F13a1* | 7 | 0.5686 | 4.66E-35 | 9.31E-32 |
| *Cebpd* | 7 | 0.5660 | 3.35E-24 | 6.69E-21 |
| *Rplp0* | 7 | 0.5606 | 3.73E-60 | 7.46E-57 |
| *Mndal* | 7 | 0.5365 | 8.47E-38 | 1.69E-34 |
| *Tifab* | 7 | 0.5344 | 1.24E-43 | 2.47E-40 |
| *Snrpg* | 7 | 0.5342 | 2.82E-42 | 5.63E-39 |
| *Hsph1* | 7 | 0.5308 | 8.51E-24 | 1.70E-20 |
| *St8sia4* | 7 | 0.5231 | 7.40E-40 | 1.48E-36 |
| *Fos* | 7 | 0.5207 | 1.29E-24 | 2.57E-21 |
| *Rpl10a* | 7 | 0.5131 | 1.23E-53 | 2.46E-50 |
| *Pmaip1* | 7 | 0.5113 | 2.00E-27 | 4.01E-24 |
| *Rsad2* | 8 | 2.2403 | 1.13E-40 | 2.27E-37 |
| *Ifit1* | 8 | 1.8001 | 1.76E-33 | 3.52E-30 |
| *Slfn5* | 8 | 1.7859 | 1.31E-44 | 2.62E-41 |
| *Ifit2* | 8 | 1.7750 | 1.59E-46 | 3.18E-43 |
| *Cxcl10* | 8 | 1.6652 | 1.13E-16 | 2.27E-13 |
| *Isg15* | 8 | 1.6406 | 1.34E-38 | 2.67E-35 |
| *Ifit3* | 8 | 1.5713 | 2.01E-44 | 4.02E-41 |
| *Irf7* | 8 | 1.4419 | 9.31E-42 | 1.86E-38 |
| *Ifi204* | 8 | 1.4142 | 8.64E-41 | 1.73E-37 |
| *Ccl12* | 8 | 1.4086 | 7.80E-11 | 1.56E-07 |
| *Ifi203* | 8 | 1.4036 | 5.78E-39 | 1.16E-35 |
| *Ms4a4c* | 8 | 1.3922 | 3.74E-26 | 7.49E-23 |
| *Cmpk2* | 8 | 1.3854 | 5.52E-39 | 1.10E-35 |
| *Mndal* | 8 | 1.3672 | 3.10E-40 | 6.20E-37 |
| *Parp14* | 8 | 1.2067 | 8.13E-32 | 1.63E-28 |
| *Plac8* | 8 | 1.1690 | 1.20E-12 | 2.40E-09 |
| *Ifi47* | 8 | 1.1386 | 3.80E-41 | 7.60E-38 |
| *Ifih1* | 8 | 1.0496 | 1.96E-37 | 3.92E-34 |
| *Fcgr1* | 8 | 0.9849 | 8.68E-30 | 1.74E-26 |
| *Ifi211* | 8 | 0.9626 | 1.11E-33 | 2.23E-30 |
| *Bst2* | 8 | 0.9218 | 1.22E-23 | 2.44E-20 |
| *Irgm1* | 8 | 0.9129 | 9.51E-33 | 1.90E-29 |
| *Ifi27l2a* | 8 | 0.9062 | 9.50E-14 | 1.90E-10 |
| *Pnp* | 8 | 0.8865 | 1.54E-27 | 3.08E-24 |
| *Ly6c2* | 8 | 0.8734 | 1.30E-12 | 2.60E-09 |
| *Gbp2* | 8 | 0.8161 | 1.08E-26 | 2.16E-23 |
| *Usp18* | 8 | 0.8003 | 5.67E-30 | 1.13E-26 |
| *Fgl2* | 8 | 0.7991 | 1.53E-18 | 3.06E-15 |
| *Ifi205* | 8 | 0.7873 | 1.76E-37 | 3.52E-34 |
| *Tent5a* | 8 | 0.7341 | 4.04E-17 | 8.08E-14 |
| *Ccl8* | 8 | 0.7159 | 4.31E-09 | 8.63E-06 |
| *Ms4a6c* | 8 | 0.7002 | 1.00E-18 | 2.01E-15 |
| *Hspa1a* | 8 | 0.6603 | 1.80E-08 | 3.60E-05 |
| *Oasl1* | 8 | 0.6536 | 2.00E-19 | 4.00E-16 |
| *Ccl3* | 8 | 0.6469 | 7.56E-07 | 0.001513 |
| *Jun* | 8 | 0.6442 | 3.11E-09 | 6.21E-06 |
| *Dnajb1* | 8 | 0.6303 | 1.76E-09 | 3.53E-06 |
| *Tmsb10* | 8 | 0.6096 | 2.62E-11 | 5.24E-08 |
| *AW112010* | 8 | 0.5992 | 1.28E-05 | 0.025594 |
| *Ifi207* | 8 | 0.5948 | 6.47E-14 | 1.29E-10 |
| *Ms4a6b* | 8 | 0.5892 | 7.65E-16 | 1.53E-12 |
| *Ifit3b* | 8 | 0.5711 | 3.95E-19 | 7.90E-16 |
| *Tmpo* | 8 | 0.5605 | 5.57E-07 | 0.001113 |
| *Ly86* | 8 | 0.5325 | 8.08E-15 | 1.62E-11 |
| *Dek* | 8 | 0.5053 | 1.31E-14 | 2.63E-11 |
